# Modeling and structural analysis of PA clan serine proteases

**DOI:** 10.1186/1756-0500-5-256

**Published:** 2012-05-24

**Authors:** Aparna Laskar, Euan J Rodger, Aniruddha Chatterjee, Chhabinath Mandal

**Affiliations:** 1 Indian Institute of Chemical Biology (CSIR Unit, Government of India), Kolkata, West Bengal, 700032, India; 2 Department of Pathology, Dunedin School of Medicine, University of Otago, Dunedin, 9054, New Zealand; 3 National Research Centre for Growth and Development, Auckland, New Zealand

**Keywords:** Serine protease, PA clan, Homology, Threading, Modeling

## Abstract

**Background:**

Serine proteases account for over a third of all known proteolytic enzymes; they are involved in a variety of physiological processes and are classified into clans sharing structural homology. The PA clan of endopeptidases is the most abundant and over two thirds of this clan is comprised of the S1 family of serine proteases, which bear the archetypal trypsin fold and have a catalytic triad in the order Histidine, Aspartate, Serine. These proteases have been studied in depth and many three dimensional structures have been experimentally determined. However, these structures mostly consist of bacterial and animal proteases, with a small number of plant and fungal proteases and as yet no structures have been determined for protozoa or archaea. The core structure and active site geometry of these proteases is of interest for many applications. This study investigated the structural properties of different S1 family serine proteases from a diverse range of taxa using molecular modeling techniques.

**Results:**

Our predicted models from protozoa, archaea, fungi and plants were combined with the experimentally determined structures of 16 S1 family members and used for analysis of the catalytic core. Amino acid sequences were submitted to SWISS-MODEL for homology-based structure prediction or the LOOPP server for threading-based structure prediction. Predicted models were refined using INSIGHT II and SCRWL and validated against experimental structures. Investigation of secondary structures and electrostatic surface potential was performed using MOLMOL. The structural geometry of the catalytic core shows clear deviations between taxa, but the relative positions of the catalytic triad residues were conserved. Some highly conserved residues potentially contributing to the stability of the structural core were identified. Evolutionary divergence was also exhibited by large variation in secondary structure features outside the core, differences in overall amino acid distribution, and unique surface electrostatic potential patterns between species.

**Conclusions:**

Encompassing a wide range of taxa, our structural analysis provides an evolutionary perspective on S1 family serine proteases. Focusing on the common core containing the catalytic site of the enzyme, this analysis is beneficial for future molecular modeling strategies and structural analysis of serine protease models.

## Background

Serine proteases represent over a third of all known proteolytic enzymes and are implicit in a wide range of physiological processes including digestion, immunity, blood clotting, fibrinolysis, reproduction and protein folding [1]. The proteolytic mechanism of these proteases involves nucleophilic attack of the carbonyl atom of the substrate peptide bond by a catalytic serine (Ser) residue in the active site of the enzyme. In addition to the nucleophilic Ser residue, this reaction is dependent on two other amino acids in the catalytic site, Histidine (His) and an Aspartate (Asp) that together form what is referred to as the catalytic triad (or a dyad in some cases) [2]. The presence of this catalytic triad in at least four distinct protein folds indicates evolutionary success in four different contexts [3].

The MEROPS classification system (http://merops.sanger.ac.uk/) has grouped proteases into clans that typically have structural homology and/or the same linear order of catalytic triad residues [4]. Of all serine proteases, the PA clan of endopeptidases is the most abundant and has been studied the most in-depth. Although most members of this clan utilize a nucleophilic Ser residue (S sub-clan), there are several viral PA proteases that alternatively use a nucleophilic cysteine (Cys) residue (C sub-clan) [5]. However, this study focuses solely on the PA clan serine proteases and more specifically members of the S1 family that bear the archetypal trypsin fold. Although extensively distributed in nature, clan PA proteases are highly represented in eukaryotes – vertebrates in particular have a vast array of proteases that are involved in a variety of extracellular processes [6]. Most clan PA proteases have trypsin-like substrate specificity, cleaving the polypeptide substrate on the carboxyl side of an arginine (Arg) or lysine (Lys) amino acid [7]. Nucleophilic attack by the Ser195 (standard chymotrypsin numbering) hydroxyl group on the carbonyl of the peptide substrate initiates the proteolytic mechanism. This reaction is catalyzed by the His57 acting as a general base, which itself is supported by a hydrogen bond to Asp102. The resulting tetrahedral intermediate is stabilized by Gly193 and Ser195, which contribute to a positively charged pocket known as the oxyanion hole. This tetrahedral intermediate breaks down to an acylenzyme intermediate, followed by the formation of a second tetrahedral intermediate. With the protonation of Ser195 by His57, the second tetrahedral intermediate breaks down and the carboxyl terminus of the substrate is released [2].

The S1 proteases are comprised of 2 β-barrels that align asymmetrically in a classical Greek key formation, bringing the catalytic residues together at their interface. The His57 and Asp102 reside in the N-terminal β-barrel with the nucleophilic Ser195 and oxyanion hole generated by the C-terminal β-barrel [8]. Many of the trypsin-like proteases are produced as an inactive zymogen precursor protein [9]. Cleavage of the proprotein precursor from the N terminus and subsequent conformational change of the tertiary structure is required for enzyme activation. In the case of trypsin, this regulatory mode of activation prevents autodegradation of the pancreas where it is produced, but allows efficient activity in the small intestine where it is activated by enteropeptidase and further trypsin molecules are activated by autocatalysis [10]. In blood coagulation and complement activation, serine protease zymogens are sequentially activated in a cascade pathway, which eventually generates effector molecules by limited proteolysis. High specificity of their catalytic domains, interactions among the regulatory regions, and efficient removal of active serine proteases by irreversible protease inhibitors ensure local, transient reactions to physiological or pathological cues [11,12]. The S1 proteases have numerous functions including intestinal digestion (eg. trypsins, chymotrypsins, elastases), blood coagulation (eg. thrombin, coagulation factors), immunity (eg. complement factors, tryptases in secretory granules of mast cells, granzymes of cytotoxic cells) and homeostatic regulation (eg. kallikreins) [1].

This study investigates the structural properties of different S1 family serine proteases from a diverse range of taxa using molecular modeling techniques. Although the catalytic core geometry shows evolutionary divergence between taxa, the relative positions of the catalytic triad residues were conserved, as were other highly conserved residues that possibly provide stabilization. There was also large variation in secondary structure features outside the core, the overall amino acid distribution, and surface electrostatic potential patterns between species.

## Methods

Structural data for 3 bacterial, 1 fungal, and 12 animal PA clan serine protease structures (Table 1) were obtained from the Protein Data Bank (PDB, http://www.rcsb.org/pdb). Our in-house modeling software package MODELYN [13] was developed to perform customized molecular editing and *in silico* structural analysis. It has a set of powerful menus for batch processing commands leading to automated implementation of complicated tasks, including complete model building based on sequence homology and batch processing of replacement mutations. ANALYN [13] is an ancillary protein sequence analysis program that assists MODELYN by analyzing homologous sequences and formulating the strategy for model building. In addition to the experimental structures, amino acid sequences of PA serine proteases (Table 1) for 1 protozoan (*Plasmodium falciparum*), 1 archaeon (*Pyrococcus furiosus*), 1 fungus (*Neurospora crassa*) and 1 plant (*Arabidopsis thaliana*) were obtained from the MEROPS protease database (http://merops.sanger.ac.uk) in FASTA format [4]. Sequences were initially submitted to SWISS-MODEL for homology-based structure prediction [14]. If this analysis was unsuccessful (due to less than 35% sequence similarity with known experimental structures), these sequences were submitted to the LOOPP server [15] for threading based structure prediction as previously described [16]. This analysis reported a ranked list of possible structure predictions for each of the protease sequences, including match scores, sequence identity (%) and the extent of sequence coverage (%). Predicted structures were superposed with respect to a selected set of Cα atoms and a suitable starting scaffold was determined. Root mean square deviation (RMSD) values helped to identify the common segments, corresponding to the structurally conserved regions. The starting structures were refined using the DISCOVER and ANALYSIS modules within the software package Insight II [17] through energy minimization and molecular dynamics. The side chains were regenerated using SCRWL [18] and the overall structure was energy minimized. The SCWRL software package is used for prediction of protein side-chains of a fixed backbone, using graph theory to solve the combinatorial problem. PROCHECK was used to check the distribution of φ-ψ dihedral angles and identify Ramachandran outliers [19]. The CHARMm module within InsightII was used to apply dihedral constraints in these segments. MOLPROBITY [20] and MODELYN were used to validate the structural models against experimental structure data. MOLPROBITY provides all-atom contact analysis and gives quantitative information on the steric interactions (H-bond and van der Waals contacts) at the interfaces between components. This program is widely used for quality validation of three-dimensional (3D) protein structures by measuring deviations of bond lengths, bond angles from standard values, overall atom clashscores and rotamer outliers. MODELYN was used to analyze other structural parameters, including the distance between Cα atoms of the catalytic triad. Verify3D [21], ProSA [22] and ERRAT [23] were also used to further assess the quality of the protease models. Verify3D analyzes the compatibility of the model against its own amino acid sequence. The Verify3D score (the sum of scores for individual residues using a 21-residue sliding window) is normalized to the length of the sequence: log_2_(Verify3D score/L^2^) [24]. ProSA calculates an overall quality score (Z score) of a model in comparison to a range of characteristics expected for native protein structures. ERRAT analyzes the statistics of non-bonded interactions between different atom types (9-residue sliding window) and provides an overall quality factor that is expressed as the percentage of the protein for which the calculated error value falls below the 95% threshold. The ribbon structure and electrostatic potential surface of the structures were determined by MOLMOL [25]. To determine sequence conservation between species, CLUSTALW [26] was used for multiple sequence alignment. For each sequence, PEPSTATS [27] was used to determine the molar percentage of each amino acid physico-chemical class.

**Table 1:** Experimental structures and predicted structures of PA serine proteases across different taxa

**Species**	**Structure**	**MEROPS ID**
**Bacteria**		
*Achromobacter lyticus*	PBD: 1ARC-A	MER000277
*Staphylococcus aureus*	PBD: 1QY6-A	MER000264
*Streptomyces griseus*	PBD: 1SGC-A	MER000251
**Protozoa**		
*Plasmodium falciparum*	**PMDB: PM0075793**	MER024901
**Archaea**		
*Pyrococcus furiosus*	**PMDB: PM0075794**	MER017398
**Fungi**		
*Fusarium oxysporum*	PBD: 1TRY-A	MER000073
*Neurospora crassa*	**PMDB: PM0075795**	MER028331
**Plantae**		
*Arabidopsis thaliana*	**PMDB: PM0075796**	MER016541
**Animalia**		
*Bos taurus*	PBD: 1EKB-B	MER000207
	PBD: 1JRS-A	MER000024
*Eisenia fetida*	PBD: 1M9U-A	MER011050
*Homo sapiens*	PBD: 1SGI-B	MER000188
	PBD: 1A0L-A	MER000136
	PBD: 1ABJ-H	MER000188
	PBD: 2ANY-A	MER000203
*Mus musculus*	PBD: 1AO5-A	MER000103
*Rattus rattus*	PBD: 1DPO-A	MER000030
*Salmo salar*	PBD: 1BIT-A	MER000035
*Solenopsis invicta*	PBD: 1EQ9-A	MER027244
*Trimeresurus stejnejer*	PBD: 1BQY-A	MER002805

## Results and Discussion

### Modeling of protease structures

The protozoan protease from *P. falciparum* was the only sequence that had significant homology with proteases of known experimental structure for successful structure prediction using SWISS-MODEL. The homology model was essentially built on the structures 1L1J (a heat shock protease from the hyperthermophilic bacterium *Thermotoga maritime*) and 2AS9 (a splC protease from the bacterium *Staphylococcus aureus*), with sequence identity ranging from 29 to 38% (Table 2). Homology-based structure prediction for the *P. furiosus*, *N. crassa* and *A. thaliana* proteases was unsuccessful due to insufficient sequence similarity with known experimental structures. The sequences of these proteases were then submitted to the LOOPP server for threading-based structure prediction, which yielded a list of 5 different experimental structures that matched to each of the sequences. The best matched structures for each showed high confidence scores ranging from 3.1 to 6.4 and sequence identity ranging from 24 to 44%, with best length coverage between 92 and 95%. For *P. furiosus* (Table 3), the matched structures were superposed with respect to a selected set of Cα atoms (43% superposition), with the structure 1GBI (an α-lytic protease from the proteobacterium *Lysobacter enzymogenes*) having the best score of 3.41 (RMSD values were between 0.357 and 0.563 Å, which helped to identify common segments corresponding to structurally conserved regions). From these superposed structures, the variable loop regions were identified on the starting scaffold derived from 1GBI. For *N. crassa* (Table 3), structures were superposed with respect to selected Cα atoms (39%) with the structure 1VCW (a degS protease from the bacterium *Escherichia coli*) having the highest score of 3.08 (RMSD values between 0.439 and 0.724 Å). For *A. thaliana* (Table 3), structures were superposed with respect to selected Cα atoms (48%), with the structure 1L1J having the best score of 6.4 (RMSD values were between 0.392 and 0.537 Å). Structural refinement using Insight II and SCRWL is provided in detail as additional information, including the refined energy status for each structural model (see Additional file 1: Table S1, Table S2, Table S3 and Table S4). PROCHECK was used to measure the overall backbone conformations of the predicted structures and identify Ramachandran outliers. The CHARMm module of Insight II was used to apply dihedral constraints in these segments (Table 4; see Additional file 1: Figure S1, Figure S2, Figure S3 and Figure S4). The general structural parameters of the refined model, such as deviations of bond lengths, bond angles from standard values, overall atom clashscores (overlaps >0.4 Å) and rotamer outliers (first two χ angles >20° from its nearest associated rotamer) were compared to experimental structure data using MOLPROBITY and MODELYN. This analysis indicated that the general structural parameters of experimental and predicted structures were comparable (Table 5). Further validation using Verify3D and ProSA gave good scores for overall model quality (Table 5). However, the ERRAT validation of the *P. falciparum* and *N. crassa* protease models indicated regions where the calculated errors were higher than expected, which decreased the overall quality score of these models (Table 5). In both cases, the low quality regions in the *P. falciparum* (Leu377-Asp387) and *N. crassa* (Ala168-Arg178) models were possibly due to steric clashes created by Phe379 (*P. falciparum*), Arg173 (*N. crassa*) and others. Significantly, these regions were not within close proximity (< 6 Å) of the catalytic site.

**Table 2:** **SWISS MODEL homology results of*Plasmodium falciparum*PA serine protease target sequence with known PDB structures**

**PDB ID**	**Resolution Å**	**R-value**	**Score (bits)**	**Expect value**	**AA identity (%)**
1L1JB	2.80	0.228	55.5	5 × 10^-9^	38
1L1JA	2.80	0.228	55.5	5 × 10^-9^	38
2AS9B	1.70	0.213	41.2	8 × 10^-5^	29
2AS9A	1.70	0.213	41.2	8 × 10^-5^	29

**Table 3:** **LOOPP server results for secondary structure matches of*Pyrococcus furiosus*,*Neurospora crassa*and*Arabidopsis thaliana*PA serine protease target sequence with known PDB structures**

**PDB ID**	**Secondary structure**			**Score**	**Sequence identity (%)**	**Length (%)**
	Helical structure (%)	Extended (%)	Loops /Other (%)			
***P. furiosus***						
Target	2.70	31.76	65.54	-	-	-
1GBI	0.00	52.41	47.59	3.410	27.14	94.59
1SSX	0.00	55.84	44.16	3.394	27.14	94.59
1GBM	0.00	55.17	44.83	3.357	27.14	94.59
1BOQ	0.00	52.41	47.59	3.343	27.14	94.59
1GBD	0.00	55.17	44.83	3.292	27.14	94.59
***N. crassa***						
Target	0.65	40.00	59.35	-	-	-
1VCW	2.60	33.77	63.64	3.078	23.87	93.55
1L1J	1.94	31.07	66.99	2.863	28.39	96.13
1TE0	2.74	32.19	65.07	2.742	24.66	87.74
1SOZ	0.00	33.51	66.49	2.535	22.73	93.55
1SOT	2.63	33.55	63.82	2.511	24.50	90.97
***A. thaliana***						
Target	0.00	38.60	61.40	-	-	-
1L1J	2.34	32.71	64.95	6.423	44.44	92.40
1VCM	3.03	35.76	61.21	6.247	42.69	92.40
1TE0	3.03	33.33	63.64	6.134	42.11	92.40
1Y8T	5.03	39.11	55.87	5.739	44.44	91.81
1SOZ	0.51	34.52	64.97	5.315	42.69	92.40

**Table 4:** **Backbone refinement of the modeled PA proteases from*Plasmodium falciparum*,*Pyrococcus furiosus, Neurospora crassa*and*Arabidopsis thaliana***

**Structural model**	**φ-ψ distribution in the regions of Ramachandran’s plot**			
	**Number of residues (percentage)**			
	Most favoured	Additional allowed	Generously allowed	Disallowed
***P. falciparum***				
Before backbone refinement	89(76.1%)	21(17.9%)	4(3.4%)	3(2.6%)
After backbone refinement	84(71.9%)	33(28.2%)	0 (0.0%)	0 (0.0%)
***P. furiosus***				
Before backbone refinement	62(56.9%)	40(36.7%)	1(0.9%)	6(5.5%)
After backbone refinement	84(71.8%)	33(28.2%)	0 (0.0%)	0 (0.0%)
***N. crassa***				
Before backbone refinement	65(52.0%)	50(40.4%)	4(3.4%)	3(2.6%)
After backbone refinement	69(55.5%)	56(44.8%)	0 (0.0%)	0 (0.0%)
***A. thaliana***				
Before backbone refinement	82(60.7%)	45(32.6%)	7(4.4%)	3(2.2%)
After backbone refinement	86(63.7%)	49(36.3%)	0 (0.0%)	0 (0.0%)

**Table 5:** **Structural validation of the modeled PA proteases from*Plasmodium falciparum*,*Pyrococcus furiosus, Neurospora crassa*and*Arabidopsis thaliana***

**Structural model**	**All atom clashscore (No/1000 atoms)**	**Rotamer outliers (%)**	**RMSD of bond Length (Å)**	**RMSD of bond angle (Degree)**
X-ray structure (1L1J)	4.33	7.49	0.029	2.74
Homology model of *P. falciparum* protease	1.86	5.26	0.030	3.14
X-ray structure (1GBI)	10.14	3.53	0.019	3.25
Threading model of *P. furiosus* protease	15.00	2.63	0.019	3.21
X-ray structure (1VCW)	3.23	4.58	0.024	3.91
Threading model of *N. crassa* protease	5.38	8.47	0.020	3.37
X-ray structure (1L1J)	4.33	7.49	0.029	2.74
Threading model of *A. thaliana* protease	11.50	8.79	0.018	3.31
	**Average Verify3D-1D**	**Normalized 3D Profile**	**ProSA**	**ERRAT**
	**score**	**score (log**_**2**_**(Verify3D/L**^**2**^**)**	**Z-score**	**quality Factor (%)**
X-ray structure (1L1J)	0.46	-10.95	-8.43	79.4
Homology model of *P. falciparum* protease	0.22	-9.28	-3.24	61.8
X-ray structure (1GBI)	0.48	-8.93	-6.73	81.6
Threading model of *P. furiosus* protease	0.19	-9.52	-3.27	71.2
X-ray structure (1VCW)	0.38	-12.80	-7.73	80.6
Threading model of *N. crassa* protease	0.24	-9.32	-3.81	52.6
X-ray structure (1L1J)	0.46	-10.95	-8.43	79.4
Threading model of *A. thaliana* protease	0.27	-9.33	-4.75	87.6

### Catalytic Core Geometry

**Figure 1:**
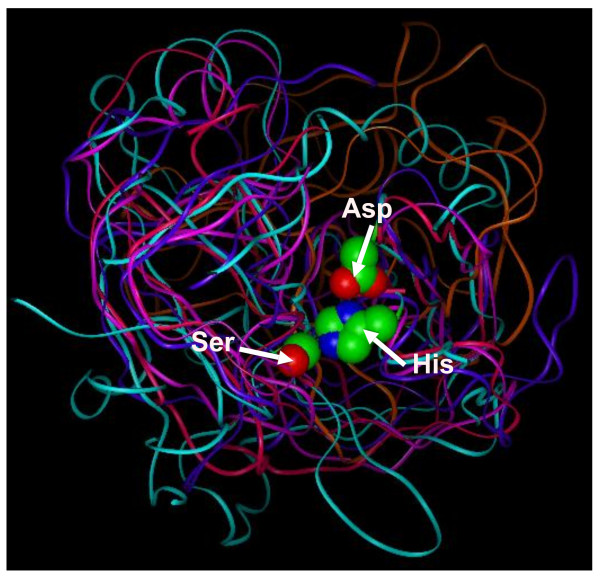
**Superposed structures of X-ray and modeled structures of the selected proteases of the PA clan.** Structures of the protozoan (*Plasmodium falciparum*, magenta), archaeon (*Pyrococcus furiosus*, cyan), fungus (*Neurospora crassa*, purple) and plant (*Arabidopsis thaliana*, fuchsia pink) PA proteases were superposed with the human x-ray structure (1SGI, *Homo sapiens*, orange). The catalytic triad residues (His, Asp, Ser) are shown in ball and stick models.

**Figure 2:**
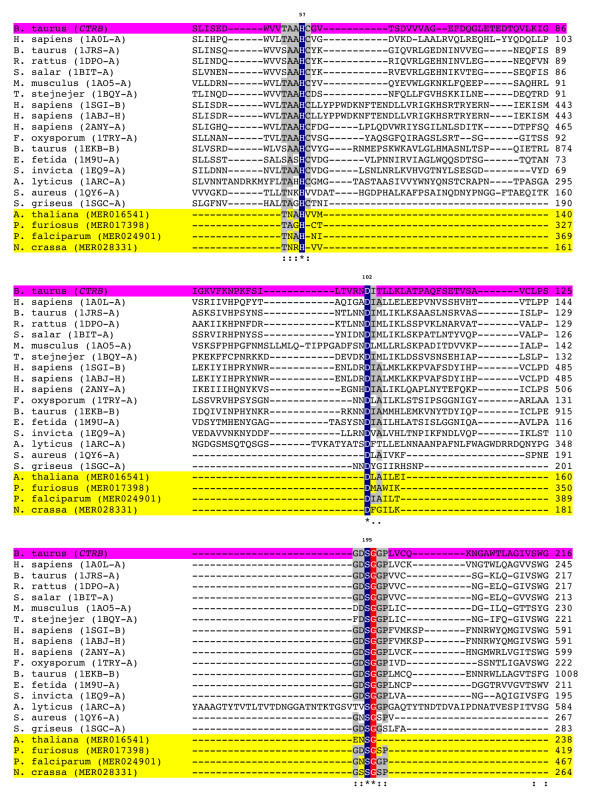
**Multiple amino acid sequence alignment of PA serine proteases.** CLUSTALW was used to align amino acid sequences of PA serine proteases for which their structures were determined experimentally or predicted computationally (highlighted in yellow). Bovine chymotrypsin B (*CTRB*, highlighted in magenta) is used as a standard reference for residue numbering. Only the regions showing the conserved catalytic residues His (H), Asp (D) and Ser (S) are shown. Amino acid residues with 100% conservation (*) between aligned sequences are either highlighted in blue (catalytic residues) or red (other). Other residues showing high (**:**) conservation (highlighted in gray) or medium (**.**) conservation are also indicated.

Superposition of the *P. falciparumP. furiosusN. crassa* and *A. thaliana* PA proteases on the representative 1SGI protease structure found that 13 to 20% of the Cα atoms superposed with a RMSD below 2Å (Table 6). In comparison, the animal proteases had 41 to 46% of the Cα atoms superposed with a RMSD below 0.8Å and the bacterial proteases of this clan had 10 to 19% of the Cα atoms superposed with a RMSD below 1Å. The superposed structures have a common core structure with large variation in loops outside the core (Figure 1). The Cα atom distances of Asp to His, His to Ser and Asp to Ser averaged over the experimentally determined structures were 6.4 ± 0.01, 8.4 ± 0.03 and 9.8 ± 0.02 Å, respectively (Table 6). The small standard deviations (SDs) indicated that the structural environment around the catalytic triad was highly conserved. Averaged over the predicted structures, the Cα atom distances between the catalytic triad residues were 6.5 ± 0.06, 8.9 ± 0.19 and 10.1 ± 0.14 Å respectively, in good agreement with the values averaged over the experimental structures. Multiple sequence alignment (Figure 2) confirmed sequence conservation of the catalytic triad residues at His57, Asp102, and Ser195 (chymotrypsin numbering). Other highly conserved amino acids have been described, including Thr54, Ala56 and Ser214, which stabilize the catalytic triad through a network of additional H-bonds [1]. These residues were highly conserved showing the occupancy percentage of 76%, 71% and 71%, respectively, among the sequences analyzed. In conjunction with the catalytic Ser195, the Gly193 residue (which was conserved in 81% of the sequences analyzed) is known to generate a positively charged pocket within the active site known as the oxyanion hole. Through intramolecular electrostatic interactions, Asp194 (71% conservation) is known to stabilize both the oxyanion hole and the substrate binding site [1]. In addition, other highly conserved amino acids such as Ala55 (81%), Cys58 (71%), Gly196 (100%), Gly197 (86%), and Pro198 (90%) were in close proximity to the catalytic residues.As confirmed in other serine proteases [28,29], such residues may confer stabilization of the catalytic site via a hydrogen-bonding interaction or via a disulfide bond in the case of the Cys residue (see Additional file 1: Figure S5, Tables S5 and Table S6). This analysis incorporates an evolutionarily diverse range of PA serine proteases and it indicates that although the core structures deviated considerably during evolution, the relative positions of the catalytic triad Cα atoms maintained very close relative distances and were stabilized by other highly conserved residues.

**Table 6:** Structural parameters of experimentally determined and predicted 3D structures of PA serine proteases

**ID**	**Taxa**	**Species**	**Superposed of AA %**	**RMSD Å**	**Distances between the catalytic triad Å**		
1ARC-A	Bacteria	*A.lyticus*	10.6	0.932	6.4	8.2	9.7
1QY6-A	Bacteria	*S. aureus*	16.2	0.753	7.1	8.4	9.9
1SGC-A	Bacteria	*S. griseus*	19.3	0.744	6.2	8.5	9.8
1TRY-A	Fungi	*F. oxysporum*	41.6	0.493	6.2	8.4	10.1
1EKB-B	Animalia	*B. taurus*	41.3	0.744	6.4	8.0	9.3
1JRS-A	Animalia	*B. taurus*	45.3	0.642	6.5	8.4	10.3
1M9U-A	Animalia	*E. fetida*	41.9	0.768	6.5	8.5	10.2
1SGI-B	Animalia	*H. sapiens*	100	0.000	6.4	8.4	10.3
1A0L-A	Animalia	*H. sapiens*	42.3	0.552	6.4	8.3	10.3
1ABJ-H	Animalia	*H. sapiens*	100	0.424	6.6	8.1	9.3
2ANY-A	Animalia	*H. sapiens*	42.4	0.541	6.3	8.3	9.8
1AO5-A	Animalia	*M. musculus*	44.1	0.552	6.6	8.2	9.7
1DPO-A	Animalia	*R. rattus*	45.5	0.652	6.3	7.6	9.8
1BIT-A	Animalia	*S. salar*	46.4	0.610	6.3	9.9	9.9
1EQ9-A	Animalia	*S. invicta*	44.6	0.593	6.3	8.1	10.1
1BQY-A	Animalia	*T. stejnejer*	41.5	0.645	6.4	8.3	9.7
Mean ± SD of the Cα distances between the triad residues					6.4±0.01	8.4±0.03	9.8±0.02
							
**PM0075793**	Protozoa	*P. falciparum*	15.0	1.003	6.2	8.4	9.9
**PM0075794**	Archaea	*P. furiosus*	22.5	0.756	6.5	8.3	9.7
**PM0075795**	Fungi	*N. crassa*	13.0	1.311	6.4	9.4	10.8
**PM0075796**	Plantae	*A. thaliana*	16.4	1.761	6.7	9.6	10.1
Mean ± SD of the Cα distances between the triad residues					6.5±0.06	8.9±0.19	10.1±0.14

### Structural analysis

**Figure 3:**
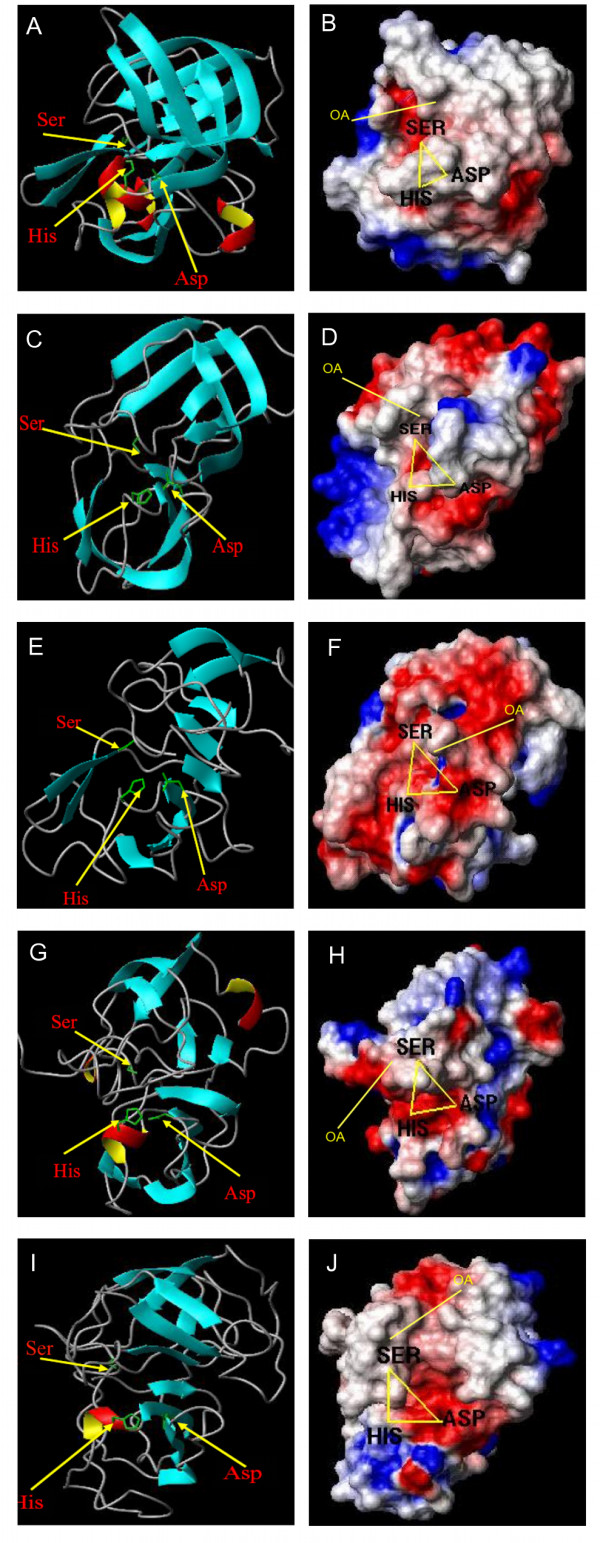
**A representative X-ray PA protease structure from*Streptomyces griseus*and modeled PA protease structures from*Plasmodium falciparum, Pyrococcus furiosus, Neurospora crassa and Arabidopsis thaliana*.** Ribbon models of *S. griseus*, 1SGC (**A**), *P. falciparum* (**C**)*, P. furiosus* (**E**)*, N. crassa* (**G**) *and A. thaliana* (**I**) PA protease structures show β-sheets with an arrow directed to the C-terminus (light blue), α-helices (red and yellow), turn/loops (gray), and catalytic triad residue side chains (green sticks). Surface electrostatic potential model of *S. griseus*, 1SGC (**B**), *P. falciparum* (**D**)*, P. furiosus* (**F**)*, N. crassa* (**H**) *and A. thaliana* (**J**) PA protease structures show electronegative (red), electropositive (blue) and electroneutral (white) amino acid side chains. The estimated position of the oxyanion hole (OA) is also indicated.

The S1 family of PA proteases is typically comprised of 2 β-barrels that align asymmetrically in a classical Greek key formation, bringing the catalytic residues together at their interface [8]. Figure 3 is a representative X-ray structure of a S1 family bacterial protease (1SGC, protease A from *Streptomyces griseus*), comprising 13 β-sheets and 4 α-helices. The protease model from *P. falciparum* had 9 β-sheets, with His328 situated in a turn, Asp359 in a coil and Ser438 in a turn (Figure 3C). The surface electrostatic potentials around the catalytic site were similar to those of the 1SGC X-ray structure, showing mostly electroneutral regions with some patches of electronegative potential (Figure 3D). In comparison with the other species analyzed (see Table S7), the *P. falciparum* protease had a higher proportion (> SD of the mean) of polar residues (55%, molar percentage) and less (< SD of the mean) smaller amino acids (43%), which indicates it could favor a more hydrophilic environment. According to UniProt annotation (Q687H5), this protease is thought to be an ortholog of the *E. coli* degP protease, which is possibly involved in protein folding and is essential for growth at high temperatures [30].

The protease model from *P. furiosus* had 7 β-sheets with His286 situated in a turn, Asp320 in a coil and Ser389 in a turn (Figure 3E). The pattern of surface electrostatic potential was very different from others analyzed, with the surface containing mostly electronegative regions around the catalytic site (Figure 3F). In comparison with the other species analyzed (see Table S7), the *P. furiosus* protease had a slightly higher proportion (> SD of the mean) of aromatic residues (12%) and less (< SD of the mean) smaller amino acids (45%). These distinctive features, which have also been observed in another *P. furiosus* protease [16], may be associated with increased stabilization and hyperthermophilic adaptation. Closely packed aromatic interactions have been proposed to increase the ΔG of unfolding, thereby increasing thermal stability [31,32]. Further investigation of these properties could be utilized for protein engineering strategies.

The protease model from *N. crassa* had 6 β-sheets and 2 α-helical segments, with His120 situated in a short α-helix and the Asp151 and Ser234 residues in separate coil regions (Figure 3G). The surface electrostatic potential pattern shows the catalytic Ser residue is in an electroneutral zone whereas the His and Asp residues are in a mostly electronegative region (Figure 3H). In general, the *N. crassa* protease had a higher proportion (> SD of the mean) of acidic residues (13%) compared to the other species analyzed (see Table S7). This protease is an ortholog of the *S. cereviseae* Nma111p nuclear serine protease, which mediates apoptosis and promotes survival under heat stress [33]. Mutational analysis of the *N. crassa* protease would be useful to explore these features in this highly studied model organism.

The *A. thaliana* PA protease model had 7 β-sheets and 1 α-helix, with His99 situated in the α-helix and Asp130 and Ser208 in separate turn structures (Figure 3I). The electrostatic potentials around the His and Ser catalytic residues were mostly electroneutral with the Asp residue of the catalytic triad in a very electronegative region (Figure 3J). The *A. thaliana* protease had a higher proportion (> SD of the mean) of aromatic residues (14%) compared to other species (see Table S7). According to UniProt annotation (Q9C691), this protease is thought to be an ortholog of degP6 and like the modeled protease from *P. falciparum* it is potentially involved in protein folding and promotes growth at high temperatures [30]. *A. thaliana* is a highly studied model organism and like the *N. crassa* protease, mutational analysis of this protease would be useful to explore these features.

The pronounced differences in electrostatic surface features between the protease catalytic sites possibly have functional significance. In general, the catalytic sites were mostly electroneutral with regions that were electronegative. The *P. falciparumA. thaliana* and *N. crassa* proteases are orthologs of the oligomeric HtrA (or HtrA-like) family of serine proteases, which have a critical role in protein quality control [34,35]. Using a hold-and-cut mechanism, the PDZ domain of most HrtA complexes selectively binds small hydrophobic residues at the C-terminus of a misfolded protein substrate, which is then successively degraded in the proteolytic site [36]. It is not surprising given the variety of functions in a wide range of different organisms that most HrtA enzymes have selective substrate specificity, although often for a number of substrates [34,35]. The electronegative patches in the catalytic sites of the modeled PA proteases could facilitate this specificity by favoring positively charged C-terminal amino acid side chains at specific sites within the binding pocket. Likewise, the largely electronegative catalytic site of the *P. furiosus* protease suggests it favors a positively charged substrate. The largely electroneutral regions possibly relax the stringency of the substrate binding, allowing for a number of different protein substrates. Further investigation of substrate specificity and other properties contributing to it would be needed for functional analysis of these proteases, particularly for the *P. falciparum* protease as it could be a potential target for rational anti-malarial drug design.

The following predicted structures are available in the Protein Model Database (PMDB) (http://mi.caspur.it/PMDB/):

1. PA serine protease from *Plasmodium falciparum* (PMDB ID: PM0075793)

2. PA serine protease from *Pyrococcus furiosus* (PMDB ID: PM0075794)

3. PA serine protease from *Neurospora crassa* (PMDB ID: PM0075795)

4. PA serine protease from *Arabidopsis thaliana* (PMDB ID: PM0075796)

## Conclusions

In conjunction with 16 experimentally determined 3D protein structures, our analysis of predicted structures from a protozoan, an archaeaon, a plant and a fungus encompassed an evolutionarily diverse range of PA clan proteases. The structural geometry of the catalytic core clearly deviated considerably during evolution, but the relative positions of the catalytic triad residues were conserved and other highly conserved residues possibly provide stabilization of the core. Evolutionary divergence was also exhibited by large variation in secondary structure features outside the core, differences in overall amino acid distribution, and unique surface electrostatic potential patterns between species. These features are probably associated with environmental adaptation, subcellular localization, and the diverse functions of the different protease orthologs. Interestingly, each of the modeled proteases appear to be orthologs of heat shock proteases that are involved in protein folding and promote cell growth at high temperatures. Indeed, some of the proteases’ features are known to confer structural stability, such as a higher proportion of aromatic residues [32] or negatively charged residues around the catalytic site [37]. Further investigation of these features would be useful for protein engineering strategies and to elucidate their functional significance in each of the modeled proteases.

## Authors’ contributions

AL participated in the design of the study and carried out the modeling, structural analysis and sequence alignment. AC contributed to MODELYN and CLUSTALW analysis. EJR drafted and revised the manuscript, with contributions by AC and AL. CM participated in the design and coordination of the study. All authors read and approved the final manuscript.

## Competing Interests

The authors have no competing interests to declare.

## Supplementary Material

Additional file 1 **Figure S1. Ramachandran plot of φ-ψ dihedral angles of a modeled PA serine protease structure from*Plasmodium falciparum*before and after backbone refinement.** PROCHECK was used to check the distribution of φ-ψ dihedral angles and eliminate Ramachandran outliers in the modeled protease structure (A, before; B, after refinement). Residues whose φ-ψ pairs fell outside the most favourable (red) and additional allowed (yellow) zones are annotated in red. **Figure S2. Ramachandran plot of φ-ψ dihedral angles of a modeled PA serine protease structure from*Pyrococcus furiosus*** before and after backbone refinement. PROCHECK was used to check the distribution of φ-ψ dihedral angles and eliminate Ramachandran outliers in the modeled protease structure (A, before; B, after refinement). Residues whose φ-ψ pairs fell outside the most favourable (red) and additional allowed (yellow) zones are annotated in red. **Figure S3. Ramachandran plot of φ-ψ dihedral angles of a modeled PA serine protease structure from*Neurospora crassa*before and after backbone refinement.** PROCHECK was used to check the distribution of φ-ψ dihedral angles and eliminate Ramachandran outliers in the modeled protease structure (A, before; B, after refinement). Residues whose φ-ψ pairs fell outside the most favourable (red) and additional allowed (yellow) zones are annotated in red. **Figure S4. Ramachandran plot of φ-ψ dihedral angles of a modeled PA serine protease structure from*Arabidopsis thaliana*before and after backbone refinement.** PROCHECK was used to check the distribution of φ-ψ dihedral angles and eliminate Ramachandran outliers in the modeled protease structure (A, before; B, after refinement). Residues whose φ-ψ pairs fell outside the most favourable (red) and additional allowed (yellow) zones are annotated in red. **Figure S5. Predicted disulfide bond in Modeled PA protease structure of*Pyrococcus furiosus*(PMDB ID: PM0075794).** The ribbon model shows secondary structures (β-sheets with arrow directed to C-terminus, α-helices and turn/loops) in alternating colors and cysteine residues Cys 267 (blue) and Cys287 (red) forming a predicted disulfide bond (2.04 Å). **Table S1.** Energy parameters of modeled PA protease structure from *Plasmodium falciparum.***Table S2.** Energy parameters of modeled PA protease structure from *Pyrococcus furiosus.***Table S3.** Energy parameters of modeled PA protease structure from *Neurospora crassa.***Table S4.** Energy parameters of modeled PA protease structure from *Arabidopsis thaliana.***Table S5.** Predicted hydrogen bonds in modeled PA protease structures. **Table S6.** Disulfide bonds in close proximity to catalytic histidine residue of experimental structures and modeled structures of PA serine proteases. **Table S7.** Relative comparison of PA serine protease amino acid composition based on physico-chemical properties. Click here for file
