# Supplementary material for: Modeling and structural analysis of PA clan serine proteases
Source: BMC Res Notes. 2012 May 24;5:256. doi: 10.1186/1756-0500-5-256 (PMC3434108; doi:10.1186/1756-0500-5-256)
Supplement: Additional file 1 — Figure S1. Ramachandran plot of φ-ψ dihedral angles of a modeled PA serine protease structure fromPlasmodium falciparumbefore and after backbone refinement. PROCHECK was used to check the distribution of φ-ψ dihedral angles and eliminate Ramachandran outliers in the modeled protease structure (A, before; B, after refinement). Residues whose φ-ψ pairs fell outside the most favourable (red) and additional allowed (yellow) zones are annotated in red. Figure S2. Ramachandran plot of φ-ψ dihedral angles of a modeled PA serine protease structure fromPyrococcus furiosus before and after backbone refinement. PROCHECK was used to check the distribution of φ-ψ dihedral angles and eliminate Ramachandran outliers in the modeled protease structure (A, before; B, after refinement). Residues whose φ-ψ pairs fell outside the most favourable (red) and additional allowed (yellow) zones are annotated in red. Figure S3. Ramachandran plot of φ-ψ dihedral angles of a modeled PA serine protease structure fromNeurospora crassabefore and after backbone refinement. PROCHECK was used to check the distribution of φ-ψ dihedral angles and eliminate Ramachandran outliers in the modeled protease structure (A, before; B, after refinement). Residues whose φ-ψ pairs fell outside the most favourable (red) and additional allowed (yellow) zones are annotated in red. Figure S4. Ramachandran plot of φ-ψ dihedral angles of a modeled PA serine protease structure fromArabidopsis thalianabefore and after backbone refinement. PROCHECK was used to check the distribution of φ-ψ dihedral angles and eliminate Ramachandran outliers in the modeled protease structure (A, before; B, after refinement). Residues whose φ-ψ pairs fell outside the most favourable (red) and additional allowed (yellow) zones are annotated in red. Figure S5. Predicted disulfide bond in Modeled PA protease structure ofPyrococcus furiosus(PMDB ID: PM0075794). The ribbon model shows secondary structures (β-sheets with arrow directed to C-terminu [file 1756-0500-5-256-S1.pdf]

Additional files

Additional file 1

Structural Refinement of Modeled PA proteases

The starting structures were refined using the Insight II equipped with DISCOVER as the energy minimization and molecular dynamics module. Structural optimization involved energy minimization (100 steps each of steepest descent and conjugate gradient methods) using cff91 force-field followed by dynamics simulations. A typical dynamics run consisted of 10000 steps of one femto-second (10 picoseconds) after 1000 steps of equilibration with a conformational sampling of 1 in 10 steps at 300K. However, dynamics simulation of 100 picoseconds was also applied to certain bigger loops for proper regularization. At the end of the dynamics simulation, the conformation with lowest potential energy was picked for the next cycle of refinement using the ANALYSIS module of Insight II. This combination of minimization and dynamics were repeated until satisfactory conformational parameters were obtained. Each loop was separately regularized applying position constraints to the rest of the atoms of the protein, which were 2 amino acids away from the desired loop by energy minimization and molecular dynamics followed by evaluation of the structural parameters. SCWRL was used to regenerate the sidechains of the modeled proteases. The final structures were energy minimized 100 steps each with steepest descent and conjugate gradient methods keeping all the atoms of the protein free.

Table S1. Energy parameters of modeled PA protease structure from *Plasmodium falciparum*

// residue		bonds	angles	torsion	improper	nonBonded	electrostatic	constraint //	TOTAL
GLY	1	1.173	1.974	0.092	0.146	-16.02	136.07	0.0000 // E=	123.439
SER	2	2.551	3.556	3.426	0.135	-24.52	5.33	0.0000 // E=	-9.524
GLY	3	1.037	2.237	1.221	0.118	-17.96	37.29	0.0000 // E=	23.947
PHE	4	1.246	3.965	2.031	1.129	-65.92	-12.27	0.0000 // E=	-69.820
ILE	5	2.580	2.349	4.241	0.417	-36.27	-5.61	0.0000 // E=	-32.300
ILE	6	1.893	6.032	4.268	2.794	-26.87	9.13	0.0000 // E=	-2.755
GLU	7	1.009	7.786	10.689	1.341	-15.08	26.43	0.0000 // E=	32.174
GLY	8	0.206	1.464	2.288	0.765	-8.89	39.39	0.0000 // E=	35.223
HISA	9	1.368	28.038	5.767	2.114	-12.90	-0.72	0.0000 // E=	23.670
LEU	10	0.348	2.241	6.062	2.171	-36.34	-6.00	0.0000 // E=	-31.527
ILE	11	1.951	7.219	5.725	1.627	-29.91	-14.96	0.0000 // E=	-28.348
ILE	12	1.858	2.857	2.980	0.823	-28.33	-9.27	0.0000 // E=	-29.085
THR	13	1.465	6.923	13.557	2.249	-30.32	-40.15	0.0000 // E=	-46.274
ASN	14	1.505	7.188	3.868	0.750	-40.03	-177.05	0.0000 // E=	-203.776
ALA	15	2.953	14.623	8.695	0.396	-27.64	112.45	0.0000 // E=	111.478
HHT	16	0.146	4.954	7.425	0.000	0.00	36.60	0.0000 // E=	49.124
HISA	16	1.111	6.915	2.624	3.005	-8.71	100.53	0.0000 // E=	105.476
ASN	17	1.781	7.059	2.037	5.667	-21.88	-158.78	0.0000 // E=	-164.110
ILE	18	1.554	5.938	4.662	4.563	-14.86	8.70	0.0000 // E=	10.556
SER	19	1.136	5.369	7.065	0.150	-15.53	-0.82	0.0000 // E=	-2.627
TYR	20	1.861	5.164	4.693	1.608	-16.29	-34.02	0.0000 // E=	-36.992
SER	21	0.237	2.620	4.326	1.229	-21.99	-17.25	0.0000 // E=	-30.829
THR	22	0.590	2.413	3.859	0.344	-10.31	-16.72	0.0000 // E=	-19.826
ARG	23	1.497	3.208	5.467	0.269	-16.94	-257.12	0.0000 // E=	-263.629
ILE	24	2.683	2.426	2.370	0.743	-24.81	-9.12	0.0000 // E=	-25.702
LEU	25	1.486	6.268	3.783	0.134	-28.52	-0.72	0.0000 // E=	-17.569
ILE	26	4.119	4.828	3.194	2.046	-16.13	-8.06	0.0000 // E=	-10.007
ARG	27	2.492	6.540	6.749	0.889	-40.24	-262.60	0.0000 // E=	-286.171
LYSH	28	2.016	4.395	5.032	0.754	-45.37	-10.97	0.0000 // E=	-44.146
HISA	29	1.180	4.202	9.487	1.751	-37.89	26.77	0.0000 // E=	5.495
GLY	30	0.421	0.567	3.272	0.054	-11.63	41.83	0.0000 // E=	34.508
ASN	31	1.547	7.468	5.418	0.581	-15.98	-166.15	0.0000 // E=	-167.110
SER	32	1.169	6.505	4.507	1.128	-14.92	23.25	0.0000 // E=	21.636
GLY	33	0.736	0.440	2.499	0.162	-16.35	33.43	0.0000 // E=	20.925
LYSH	34	1.252	1.999	3.023	0.079	-29.17	1.30	0.0000 // E=	-21.511
TYR	35	1.189	4.156	3.623	2.515	-44.94	-55.16	0.0000 // E=	-88.614
GLU	36	2.229	4.544	15.427	0.889	-26.38	1.05	0.0000 // E=	-2.242
ALA	37	0.790	1.795	0.829	0.566	-24.44	-6.48	0.0000 // E=	-26.945
LYSH	38	2.677	8.241	8.954	0.256	-17.14	-15.64	0.0000 // E=	-12.647
ILE	39	4.419	3.799	3.806	1.366	-20.47	3.92	0.0000 // E=	-3.153
LEU	40	1.818	5.389	4.449	0.913	-27.12	4.43	0.0000 // E=	-10.119
TYR	41	1.898	3.062	3.662	0.493	-31.33	-51.39	0.0000 // E=	-73.598
VAL	42	3.626	5.944	2.758	4.003	-21.62	-1.93	0.0000 // E=	-7.225
ALA	43	1.169	1.771	4.992	0.270	-23.58	-12.82	0.0000 // E=	-28.197
HISA	44	0.542	6.580	6.983	0.879	-32.82	7.69	0.0000 // E=	-10.146
ASP	45	0.853	2.197	4.678	0.840	-16.96	15.40	0.0000 // E=	7.009
VAL	46	2.459	6.680	6.260	2.881	-20.79	8.80	0.0000 // E=	6.290
ASP	47	3.799	45.809	12.614	1.585	9.16	-12.58	0.0000 // E=	60.395
ILE	48	0.871	5.016	3.730	2.222	-19.51	-14.19	0.0000 // E=	-21.866
ALA	49	1.063	2.495	1.870	0.424	-29.87	-9.26	0.0000 // E=	-33.280
ILE	50	4.065	4.879	3.567	0.776	-27.67	-17.79	0.0000 // E=	-32.174
LEU	51	1.178	5.253	1.030	0.397	-47.76	-13.39	0.0000 // E=	-53.295
THR	52	0.942	2.755	6.588	0.853	-27.53	-10.28	0.0000 // E=	-26.675
THR	53	1.672	23.465	11.680	1.951	1.78	8.91	0.0000 // E=	49.457
ASP	54	0.325	6.042	3.304	1.142	-4.95	2.10	0.0000 // E=	7.962
ASP	55	0.948	3.791	54.767	2.751	-16.37	6.34	0.0000 // E=	52.229
LYSH	56	1.115	8.857	20.879	2.361	-16.61	-4.03	0.0000 // E=	12.567
THR	57	0.525	5.560	4.210	2.425	-18.14	-7.52	0.0000 // E=	-12.938
PHE	58	0.305	1.199	6.617	0.647	-17.75	5.87	0.0000 // E=	-3.113
PHE	59	0.705	5.061	8.119	1.243	-22.40	4.14	0.0000 // E=	-3.135
ASP	60	0.216	4.337	6.451	0.403	-17.28	-1.31	0.0000 // E=	-7.190
ASP	61	0.182	0.704	7.039	2.125	-24.68	7.62	0.0000 // E=	-7.015
VAL	62	0.780	5.907	4.956	2.538	-12.53	10.66	0.0000 // E=	12.314
TYR	63	0.703	3.978	56.239	9.352	-28.15	-37.69	0.0000 // E=	4.434
ALA	64	0.080	1.708	1.418	0.491	-27.64	-0.19	0.0000 // E=	-24.128
LEU	65	0.147	3.755	6.507	0.329	-28.22	-12.45	0.0000 // E=	-29.930
HISA	66	5.803	10.852	5.588	5.904	0.65	6.92	0.0000 // E=	35.715
PHE	67	64.422	344.998	20.377	104.046	163.30	42.31	0.0000 // E=	739.462
GLY	68	1.840	11.465	20.531	1.675	17.91	38.46	0.0000 // E=	91.879
ALA	69	0.769	4.647	8.020	1.009	-11.17	2.56	0.0000 // E=	5.836
LEU	70	2.130	35.129	5.881	1.383	3.23	23.47	0.0000 // E=	71.220
PRO	71	0.764	17.755	14.620	0.690	-8.44	-20.13	0.0000 // E=	5.254
SER	72	0.810	6.878	9.497	0.189	-11.69	-2.24	0.0000 // E=	3.440
LEU	73	0.480	3.267	12.787	1.567	-11.40	2.98	0.0000 // E=	9.682
LYSH	74	1.391	6.992	9.126	0.626	-31.32	-6.00	0.0000 // E=	-19.186
ASP	75	3.396	5.222	7.937	0.634	-10.60	1.27	0.0000 // E=	7.862
GLU	76	5.970	28.471	12.135	0.548	-24.67	0.28	0.0000 // E=	22.734
ILE	77	5.441	22.681	7.698	1.610	-32.23	-21.50	0.0000 // E=	-16.304
ILE	78	4.327	5.405	2.940	0.864	-24.92	-14.19	0.0000 // E=	-25.575
THR	79	3.258	3.778	4.919	1.247	-35.39	-30.48	0.0000 // E=	-52.665
ILE	80	4.448	3.979	3.483	0.729	-29.33	27.91	0.0000 // E=	11.217
GLY	81	1.332	3.244	0.083	1.492	-24.94	22.46	0.0000 // E=	3.666
TYR	82	2.420	8.838	6.404	0.496	-48.72	-15.06	0.0000 // E=	-45.621
PRO	83	0.554	17.915	19.938	0.981	-20.71	-17.35	0.0000 // E=	1.321
ALA	84	0.977	4.480	2.492	1.284	-11.40	40.17	0.0000 // E=	38.004
GLY	85	0.450	1.102	3.902	0.572	-12.21	60.13	0.0000 // E=	53.940
GLY	86	0.515	1.644	3.279	0.127	-12.84	30.73	0.0000 // E=	23.448
ASP	87	0.289	4.743	2.202	0.471	-26.70	-5.86	0.0000 // E=	-24.850
LYSH	88	0.502	2.118	6.052	0.909	-23.77	6.81	0.0000 // E=	-7.375
LEU	89	1.417	19.064	6.542	0.962	-22.19	10.47	0.0000 // E=	16.259
SER	90	0.252	10.949	19.273	0.924	-18.79	-23.36	0.0000 // E=	-10.755
VAL	91	0.639	1.166	3.332	0.675	-23.32	4.95	0.0000 // E=	-12.564
THR	92	0.435	1.783	8.057	0.974	-31.74	-26.91	0.0000 // E=	-47.404
GLU	93	0.975	2.341	3.223	0.381	-22.50	38.22	0.0000 // E=	22.640
GLY	94	0.977	1.592	0.322	0.011	-17.47	28.90	0.0000 // E=	14.339
ILE	95	2.638	4.021	4.550	1.780	-25.24	-7.87	0.0000 // E=	-20.118
VAL	96	2.098	5.259	3.881	6.590	-28.25	5.67	0.0000 // E=	-4.758
SER	97	0.964	5.896	8.959	1.006	-22.96	-10.76	0.0000 // E=	-16.894
ARG	98	2.484	8.175	7.707	0.894	-24.67	-268.36	0.0000 // E=	-273.771
ILE	99	2.253	4.052	8.681	1.851	-27.53	6.21	0.0000 // E=	-4.473
ASP	100	2.353	5.878	2.085	1.452	-20.83	-1.32	0.0000 // E=	-10.389
VAL	101	1.375	3.158	5.309	1.682	-29.13	-4.22	0.0000 // E=	-21.823
GLN	102	1.115	3.910	7.430	0.300	-36.86	-177.77	0.0000 // E=	-201.884
TYR	103	0.306	2.614	7.423	0.553	-42.31	-56.04	0.0000 // E=	-87.458
TYR	104	0.448	2.160	2.981	0.330	-44.85	-51.56	0.0000 // E=	-90.483
LYSH	105	1.357	14.028	19.466	1.657	-15.89	-4.31	0.0000 // E=	16.308
HISA	106	3.212	25.944	13.915	4.030	-13.75	11.08	0.0000 // E=	44.425
SER	107	0.587	71.718	24.465	0.227	-11.14	-25.78	0.0000 // E=	60.076
ASN	108	1.536	6.756	14.834	3.228	-16.95	-173.49	0.0000 // E=	-164.089
TYR	109	0.362	1.619	2.189	0.966	-45.09	-47.68	0.0000 // E=	-87.631
LYSH	110	5.574	18.468	13.454	0.547	-14.35	-7.60	0.0000 // E=	16.091
PHE	111	0.230	0.869	3.652	0.717	-31.95	6.80	0.0000 // E=	-19.684
LEU	112	1.250	6.197	2.684	1.545	-22.01	2.34	0.0000 // E=	-7.998
LEU	113	0.531	6.593	2.627	0.164	-42.79	-4.99	0.0000 // E=	-37.861
THR	114	0.879	5.385	9.999	1.480	-21.58	-9.68	0.0000 // E=	-13.516
GLN	115	1.519	5.013	5.019	0.614	-37.61	-181.49	0.0000 // E=	-206.942

ILE	116	1.903	4.317	5.406	2.350	-31.59	4.29	0.0000	// E=	-13.325
ASP	117	1.762	4.209	4.542	1.339	-23.44	1.09	0.0000	// E=	-10.494
ALA	118	1.411	3.018	2.048	0.027	-21.87	24.86	0.0000	// E=	9.489
PRO	119	1.742	21.173	16.628	4.890	-19.44	-35.66	0.0000	// E=	-10.673
LEU	120	1.923	3.893	2.108	0.650	-27.05	0.86	0.0000	// E=	-17.617
ASN	121	3.270	6.313	7.701	1.212	-24.63	-179.58	0.0000	// E=	-185.717
PRO	122	1.024	22.698	13.878	2.028	-15.61	11.70	0.0000	// E=	35.713
GLY	123	0.611	1.201	4.939	0.085	-19.43	28.94	0.0000	// E=	16.340
ASN	124	2.181	13.704	2.973	1.796	-39.24	-188.51	0.0000	// E=	-207.093
SER	125	1.526	1.729	2.233	0.086	-21.82	-1.81	0.0000	// E=	-18.057
GLY	126	0.814	3.499	1.309	0.376	-27.23	68.95	0.0000	// E=	47.718
GLY	127	0.244	1.638	3.344	0.386	-15.34	51.67	0.0000	// E=	41.940
PRO	128	3.139	41.645	12.576	0.600	-19.96	-32.10	0.0000	// E=	5.892
ALA	129	1.853	1.228	3.445	0.686	-35.58	-20.16	0.0000	// E=	-48.520
LEU	130	6.058	40.164	9.688	0.374	-31.09	-18.42	0.0000	// E=	6.768
VAL	131	2.693	14.937	12.119	3.692	-4.23	-9.10	0.0000	// E=	20.103
ARG	132	6.130	65.507	7.708	1.585	-1.79	-215.89	0.0000	// E=	-136.750
GLY	133	2.951	2.115	1.714	0.762	-12.28	40.49	0.0000	// E=	35.747
LYSH	134	70.535	114.694	40.086	1.966	58.86	-8.14	0.0000	// E=	278.003
VAL	135	48.193	296.793	53.665	50.548	91.59	0.11	0.0000	// E=	540.900
VAL	136	5.453	16.108	1.843	4.679	9.57	40.11	0.0000	// E=	77.754
GLY	137	1.011	3.954	4.211	0.853	-13.89	32.04	0.0000	// E=	28.179
ILE	138	3.134	5.440	8.234	2.386	-21.89	101.83	0.0000	// E=	99.139
//-------										
KJ/mol		415.064	1872.979	1086.253	339.968	-2699.08	-1965.17	0.0000	// E=	-949.983

// Computations were done in vacuo with the GROMOS96 43B1 parameters set, without reaction field.
// For more information about GROMOS96, refer to: W.F. van Gunsteren et al. (1996) in Biomolecular
// simulation: the GROMOS96 manual and user guide. Vdf Hochschulverlag ETHZ (http://igc.ethz.ch/gromos).
// When using those results, please mention that energy computations were done with the GROMOS96
// implementation of Swiss-PdbViewer.

Table S2. Energy parameters of modeled PA protease structure from *Pyrococcus furiosus*

// residue	bonds	angles	torsion	improper	nonBonded	electrostatic	constraint	//	TOTAL
GLY A 3	1.128	1.424	1.754	0.863	-5.90	187.50	0.0000	// E=	186.763
GLY A 4	1.226	1.946	3.806	0.046	-17.64	48.80	0.0000	// E=	38.176
ILE A 5	4.144	10.493	3.880	2.541	-23.69	9.51	0.0000	// E=	6.885
GLN A 6	2.223	3.497	6.098	0.326	-31.95	-164.84	0.0000	// E=	-184.642
PHE A 7	1.284	1.929	4.346	0.626	-56.42	-8.97	0.0000	// E=	-57.203
GLN A 8	2.448	8.025	2.699	0.427	-41.73	-184.68	0.0000	// E=	-212.814
VAL A 9	3.184	7.574	6.325	1.130	-27.34	12.49	0.0000	// E=	3.364
PRO A 10	1.013	19.728	22.640	1.489	-26.29	8.11	0.0000	// E=	26.690
GLY A 11	0.566	3.882	3.248	0.176	-15.01	40.99	0.0000	// E=	33.851
HISA A 12	1.230	10.562	18.010	1.160	-26.40	-2.32	0.0000	// E=	2.247
ASN A 13	2.515	5.474	8.306	0.473	-33.40	-168.83	0.0000	// E=	-185.460
TYR A 14	1.854	2.085	2.823	1.322	-38.42	-41.32	0.0000	// E=	-71.656
CYS1 A 15	2.460	7.005	19.604	0.532	-30.98	-16.92	0.0000	// E=	-18.300
THR A 16	2.479	7.559	4.734	2.830	-35.97	-10.11	0.0000	// E=	-28.483
LEU A 17	1.722	7.843	10.467	2.106	-40.25	42.32	0.0000	// E=	24.217
GLY A 18	0.372	7.602	13.736	0.464	-23.84	36.72	0.0000	// E=	35.057
PHE A 19	0.854	2.577	8.504	0.551	-49.19	9.84	0.0000	// E=	-26.859
PRO A 20	0.320	13.364	19.146	0.571	-41.01	-24.89	0.0000	// E=	-32.498
ALA A 21	0.385	1.882	2.861	0.429	-20.18	3.81	0.0000	// E=	-10.818
GLU A 22	0.496	2.386	9.377	2.340	-42.29	-7.56	0.0000	// E=	-35.255
LYSH A 23	3.028	6.553	7.258	0.845	-29.49	-6.42	0.0000	// E=	-18.225
ASN A 24	2.287	5.099	6.802	0.532	-8.19	-137.87	0.0000	// E=	-131.342
GLY A 25	0.568	3.646	2.762	0.527	-10.57	39.03	0.0000	// E=	35.966
ILE A 26	2.818	4.790	4.052	1.208	-15.84	-11.68	0.0000	// E=	-14.650
VAL A 27	2.378	3.208	3.645	1.635	-31.93	33.65	0.0000	// E=	12.585
GLY A 28	0.618	4.037	3.117	0.893	-25.46	26.31	0.0000	// E=	9.521
MET A 29	0.877	7.684	19.311	2.037	-48.42	-10.98	0.0000	// E=	-29.495
VAL A 30	2.034	3.140	3.132	1.235	-31.94	-17.16	0.0000	// E=	-39.555
THR A 31	2.084	3.338	7.059	0.884	-40.41	-23.56	0.0000	// E=	-50.609
ALA A 32	1.084	1.002	3.917	0.766	-28.90	48.38	0.0000	// E=	26.246
GLY A 33	0.339	1.746	1.594	0.250	-22.29	38.63	0.0000	// E=	20.276
HISB A 34	1.199	6.348	8.339	3.725	-24.24	-5.37	0.0000	// E=	-10.004
CYS2 A 35	1.086	4.994	9.684	0.268	-30.28	1.54	0.0000	// E=	-12.711
THR A 36	2.346	4.380	7.887	1.322	-34.62	-14.21	0.0000	// E=	-32.899
ASP A 37	2.307	6.901	3.239	0.248	-29.41	11.13	0.0000	// E=	-5.581
GLU A 38	1.651	2.240	9.359	1.813	-24.79	37.00	0.0000	// E=	27.277
GLY A 39	0.991	9.005	2.883	0.512	-14.91	38.17	0.0000	// E=	36.654
ALA A 40	2.576	6.852	1.312	0.229	-18.03	18.65	0.0000	// E=	11.588
PRO A 41	0.961	15.313	16.710	1.794	-30.58	-31.35	0.0000	// E=	-27.148
ALA A 42	0.664	0.806	3.618	0.036	-28.04	-7.59	0.0000	// E=	-30.511
TYR A 43	0.663	3.828	5.214	0.531	-60.73	-63.26	0.0000	// E=	-113.758
GLN A 44	1.969	11.532	13.018	1.365	-34.11	-142.19	0.0000	// E=	-148.422
PRO A 45	0.791	15.023	24.288	1.603	-19.80	-23.83	0.0000	// E=	-1.915
ASP A 46	2.239	13.281	6.761	0.361	-24.45	-18.66	0.0000	// E=	-20.474
THR A 47	4.097	10.678	3.809	0.890	-30.82	-20.51	0.0000	// E=	-31.858
SER A 48	1.638	2.537	4.139	2.683	-15.76	-3.92	0.0000	// E=	-8.684
ASP A 49	1.779	7.286	6.715	0.433	-11.85	35.43	0.0000	// E=	39.797
PRO A 50	0.713	24.283	20.878	2.417	-12.15	-25.43	0.0000	// E=	10.712
SER A 51	1.553	2.326	6.279	2.010	-17.23	-7.15	0.0000	// E=	-12.208
TYR A 52	4.465	35.290	2.933	11.882	-31.34	-46.44	0.0000	// E=	-23.209
TYR A 53	0.793	20.611	84.093	15.866	-27.97	-51.78	0.0000	// E=	41.607
ILE A 54	1.275	5.708	6.573	2.742	-33.68	35.82	0.0000	// E=	18.440
GLY A 55	0.106	1.749	1.469	0.066	-17.05	28.38	0.0000	// E=	14.718
ASN A 56	1.254	3.890	2.838	0.244	-39.04	-171.06	0.0000	// E=	-201.874
VAL A 57	0.949	6.559	5.591	4.215	-29.84	-6.29	0.0000	// E=	-18.823
GLU A 58	1.730	3.623	6.220	0.560	-27.28	1.22	0.0000	// E=	-13.928
ILE A 59	6.532	18.102	8.754	7.593	-8.46	3.22	0.0000	// E=	35.745
LYSH A 60	0.645	9.386	19.461	4.984	-41.61	-6.59	0.0000	// E=	-13.727
LEU A 61	0.455	2.566	2.400	0.785	-34.14	-10.84	0.0000	// E=	-38.778
TRP A 62	0.631	3.625	5.938	2.863	-44.29	-17.79	0.0000	// E=	-49.020
SER A 63	0.713	1.607	1.533	0.685	-17.41	15.34	0.0000	// E=	2.471
PRO A 64	1.318	19.207	18.767	0.587	-12.88	-23.22	0.0000	// E=	3.781
ALA A 65	0.958	1.552	2.141	0.063	-19.50	-0.30	0.0000	// E=	-15.087
GLN A 66	3.834	33.803	4.585	20.557	-16.77	-126.43	0.0000	// E=	-80.419
GLY A 67	0.736	14.139	16.280	22.113	-20.19	29.66	0.0000	// E=	62.730
ASP A 68	0.785	3.734	13.961	1.383	-36.72	-12.51	0.0000	// E=	-29.359
MET A 69	1.463	5.453	8.950	0.146	-38.36	-13.31	0.0000	// E=	-35.658
ALA A 70	0.615	2.259	4.278	0.069	-28.23	-13.75	0.0000	// E=	-34.758
TRP A 71	2.106	8.370	5.654	2.950	-51.68	-18.57	0.0000	// E=	-51.174
ILE A 72	2.520	4.214	2.780	2.747	-34.51	-13.99	0.0000	// E=	-36.235
LYSH A 73	1.886	9.835	23.176	0.387	-32.29	-12.00	0.0000	// E=	-9.005
THR A 74	2.598	4.452	2.460	0.911	-30.51	-21.93	0.0000	// E=	-42.022
THR A 75	2.119	2.073	2.324	1.237	-17.52	-28.28	0.0000	// E=	-38.049
VAL A 76	3.361	12.776	0.755	7.821	-16.50	34.71	0.0000	// E=	42.924
GLY A 77	1.434	1.668	3.018	0.046	-9.24	38.21	0.0000	// E=	35.135
VAL A 78	2.878	5.612	2.888	3.551	-10.35	1.81	0.0000	// E=	6.387
THR A 79	4.986	15.929	6.350	3.223	-16.95	18.41	0.0000	// E=	31.941
PRO A 80	1.234	15.616	19.496	0.600	-24.92	-24.43	0.0000	// E=	-12.401
LYSH A 81	1.730	7.042	13.786	0.117	-37.52	-9.24	0.0000	// E=	-24.085
VAL A 82	2.578	6.457	3.542	0.943	-31.93	-14.57	0.0000	// E=	-32.981
TYR A 83	1.321	4.964	2.946	0.363	-57.44	-16.34	0.0000	// E=	-64.188
PRO A 84	0.885	19.007	35.097	0.502	-28.42	-30.57	0.0000	// E=	-3.500
TYR A 85	1.606	5.452	3.780	2.942	-38.20	-48.35	0.0000	// E=	-72.763
PHE A 86	2.031	17.892	9.501	6.432	-21.12	-1.21	0.0000	// E=	13.527
ILE A 87	2.877	7.937	27.157	6.604	-5.60	-1.71	0.0000	// E=	37.270
ILE A 88	3.169	4.639	3.156	3.842	-12.56	1.78	0.0000	// E=	4.023
LYSH A 89	1.263	11.379	3.300	0.226	-34.53	19.39	0.0000	// E=	1.030
GLY A 90	1.084	1.656	2.011	0.012	-14.68	36.86	0.0000	// E=	26.949
TYR A 91	1.979	9.907	6.033	1.709	-37.65	-37.89	0.0000	// E=	-55.909
LYSH A 92	1.907	5.383	2.342	0.022	-24.83	15.75	0.0000	// E=	0.570
PRO A 93	0.782	19.126	22.096	7.630	-9.85	-29.10	0.0000	// E=	10.675
TYR A 94	1.017	4.422	4.801	7.568	-41.05	-55.98	0.0000	// E=	-79.223
ARG A 95	2.841	7.670	6.039	1.774	-19.52	-249.89	0.0000	// E=	-251.090
TYR A 96	1.568	6.292	3.196	2.097	-30.41	-34.07	0.0000	// E=	-51.323
GLN A 97	2.598	6.411	3.647	0.478	-26.08	-177.20	0.0000	// E=	-190.143
TYR A 98	2.524	6.536	8.089	0.490	-21.79	-46.33	0.0000	// E=	-50.476
VAL A 99	2.561	3.425	5.757	1.412	-10.19	41.64	0.0000	// E=	44.600
GLY A 100	0.738	6.995	1.863	0.593	-12.74	32.78	0.0000	// E=	30.230
SER A 101	2.043	1.987	2.318	0.389	-22.76	-26.27	0.0000	// E=	-42.293
THR A 102	2.394	3.231	1.869	1.307	-17.75	-7.12	0.0000	// E=	-16.067
VAL A 103	1.617	2.684	2.313	2.065	-32.63	-7.69	0.0000	// E=	-31.640
LEU A 104	2.267	6.963	2.834	0.943	-33.32	0.62	0.0000	// E=	-19.696
LYSH A 105	3.893	10.220	8.792	1.604	-53.72	-20.44	0.0000	// E=	-49.657
SER A 106	2.541	6.069	22.778	1.310	-31.03	35.69	0.0000	// E=	37.359
GLY A 107	1.295	2.113	2.176	0.123	-27.13	61.48	0.0000	// E=	40.064
ARG A 108	3.542	20.402	13.114	2.458	-38.19	-250.64	0.0000	// E=	-249.309
THR A 109	1.955	5.659	7.485	1.461	-22.45	23.00	0.0000	// E=	17.112
THR A 110	2.182	12.938	10.594	2.181	-16.79	45.02	0.0000	// E=	56.116
GLY A 111	0.494	2.384	2.384	0.221	-22.01	52.72	0.0000	// E=	36.190
LEU A 112	1.607	3.671	3.976	0.890	-29.61	0.01	0.0000	// E=	-19.451
THR A 113	1.846	5.346	3.330	1.293	-23.50	16.71	0.0000	// E=	5.030
GLY A 114	0.537	0.534	4.156	0.042	-11.01	69.90	0.0000	// E=	64.152
GLY A 115	0.575	0.751	1.572	0.001	-14.48	25.89	0.0000	// E=	14.299
ILE A 116	0.757	2.169	2.276	0.546	-23.48	-3.09	0.0000	// E=	-20.824
ILE A 117	0.962	1.780	8.700	0.181	-32.88	-7.50	0.0000	// E=	-28.758
LEU A 118	0.652	3.143	4.424	1.477	-28.44	12.06	0.0000	// E=	-6.685
ASP A 119	0.933	4.768	1.290	2.796	-22.56	4.62	0.0000	// E=	-8.157

ILE	A 120	0.930	5.300	4.068	1.546	-22.55	6.19	0.0000 //	E=	-4.521
SER	A 121	0.175	3.055	8.459	1.334	-22.34	-6.00	0.0000 //	E=	-15.316
THR	A 122	1.526	7.614	19.131	2.523	-26.36	-34.52	0.0000 //	E=	-30.086
SER	A 123	3.540	10.854	8.793	2.653	-7.70	-14.95	0.0000 //	E=	3.192
LEU	A 124	2.586	36.436	2.970	3.442	3.98	-1.69	0.0000 //	E=	47.724
GLU	A 125	0.697	21.172	22.412	12.800	-20.44	-1.40	0.0000 //	E=	35.244
ILE	A 126	1.783	7.681	21.718	1.228	-4.56	4.69	0.0000 //	E=	32.531
ARG	A 127	1.751	9.209	10.783	1.923	-41.34	-270.51	0.0000 //	E=	-288.192
THR	A 128	0.926	2.771	14.797	2.024	-14.11	-15.66	0.0000 //	E=	-9.252
THR	A 129	1.266	2.016	7.369	0.343	-28.57	-32.64	0.0000 //	E=	-50.220
MET	A 130	3.137	9.139	8.511	3.306	-47.94	-2.29	0.0000 //	E=	-26.137
GLU	A 131	8.868	28.028	12.655	5.823	-44.44	3.59	0.0000 //	E=	14.516
VAL	A 132	10.686	50.218	25.592	14.556	-19.56	4.54	0.0000 //	E=	86.042
ALA	A 133	1.205	3.217	2.128	2.474	-5.89	27.95	0.0000 //	E=	31.085
PRO	A 134	0.639	17.987	27.025	1.727	-29.46	13.83	0.0000 //	E=	31.742
GLY	A 135	0.327	0.473	6.345	0.527	-9.45	41.15	0.0000 //	E=	39.375
ASP	A 136	0.695	2.455	4.249	0.407	-37.25	-3.73	0.0000 //	E=	-33.175
SER	A 137	0.974	1.425	2.082	0.538	-26.52	29.27	0.0000 //	E=	7.764
GLY	A 138	0.884	8.413	2.335	1.787	-19.71	42.67	0.0000 //	E=	36.375
SER	A 139	2.304	4.013	1.045	2.805	-28.05	24.41	0.0000 //	E=	6.531
PRO	A 140	0.817	16.490	17.133	3.001	-42.71	-31.48	0.0000 //	E=	-36.747
VAL	A 141	2.126	3.870	4.229	1.062	-24.69	4.47	0.0000 //	E=	-8.933
PHE	A 142	2.496	12.244	5.340	2.358	-53.86	84.16	0.0000 //	E=	52.738
KJ/mol		253.108	1121.079	1195.992	311.554	-3750.23	-1639.93	0.0000 //	E=	-2508.426

// Computations were done in vacuo with the GROMOS96 43B1 parameters set, without reaction field.
// For more information about GROMOS96, refer to: W.F. van Gunsteren et al. (1996) in Biomolecular
// simulation: the GROMOS96 manual and user guide. Vdf Hochschulverlag ETHZ (http://igc.ethz.ch/gromos).
// When using those results, please mention that energy computations were done with the GROMOS96
// implementation of Swiss-PdbViewer.

Table S3. Energy parameters of modeled PA protease structure from *Neurospora crassa*

residue	bonds	angles	torsion	improper	nonBonded	electrostatic	constraint //	TOTAL
GLY A 2	0.828	2.503	0.307	0.414	-6.96	137.98	0.0000 // E=	135.073
PHE A 3	1.384	4.047	12.021	1.864	-55.42	5.81	0.0000 // E=	-30.300
VAL A 4	1.603	4.031	2.975	1.653	-34.29	-1.80	0.0000 // E=	-25.828
VAL A 5	0.453	4.238	1.820	2.317	-29.25	-0.96	0.0000 // E=	-21.383
ASP A 6	0.672	3.765	7.727	2.063	-28.27	-4.38	0.0000 // E=	-18.422
ALA A 7	1.389	15.357	20.211	1.958	-4.39	1.07	0.0000 // E=	35.598
GLU A 8	2.625	9.371	28.512	1.214	14.24	-11.20	0.0000 // E=	44.757
ARG A 9	1.824	4.866	11.616	1.976	-17.56	-196.17	0.0000 // E=	-193.447
GLY A 10	0.244	1.975	4.459	0.167	-20.50	51.43	0.0000 // E=	37.774
TYR A 11	0.842	3.817	8.977	1.462	-50.46	-48.14	0.0000 // E=	-83.498
ILE A 12	1.064	2.410	5.120	1.562	-38.14	-4.59	0.0000 // E=	-32.573
LEU A 13	6.036	33.493	6.067	11.065	8.33	-4.29	0.0000 // E=	60.701
THR A 14	3.064	6.124	4.266	3.373	-33.22	-31.48	0.0000 // E=	-47.877
ASN A 15	1.342	3.951	14.445	0.573	-40.33	-178.21	0.0000 // E=	-198.226
ARG A 16	2.576	6.857	1.875	1.378	-63.11	-262.59	0.0000 // E=	-313.011
HISB A 17	1.402	6.732	9.204	3.795	-11.42	-12.48	0.0000 // E=	-2.767
VAL A 18	0.401	2.030	3.176	0.862	-18.71	15.36	0.0000 // E=	3.123
VAL A 19	0.548	4.308	3.536	1.876	-19.75	37.73	0.0000 // E=	28.249
GLY A 20	0.226	2.099	10.387	2.116	-9.42	35.54	0.0000 // E=	40.951
SER A 21	1.535	6.370	9.058	0.025	-3.99	26.05	0.0000 // E=	39.048
GLY A 22	0.521	10.002	60.216	0.126	-10.51	58.91	0.0000 // E=	119.262
PRO A 23	0.788	16.362	25.468	0.225	-20.74	-21.75	0.0000 // E=	0.347
PHE A 24	1.001	4.543	4.743	0.646	-30.31	6.69	0.0000 // E=	-12.692
TRP A 25	2.019	5.147	6.556	2.277	-43.39	9.84	0.0000 // E=	-17.548
GLY A 26	1.600	2.158	3.649	0.177	-14.76	34.40	0.0000 // E=	27.225
TYR A 27	2.643	9.401	18.348	1.195	-60.24	-42.14	0.0000 // E=	-70.793
CYSH A 28	2.610	4.335	3.981	0.543	-38.43	-0.14	0.0000 // E=	-27.109
ILE A 29	3.469	6.143	7.640	1.938	-37.08	-4.93	0.0000 // E=	-22.822
PHE A 30	1.339	8.109	2.759	3.094	-32.94	12.35	0.0000 // E=	-5.293
ASP A 31	1.144	12.955	3.281	1.346	-29.40	6.12	0.0000 // E=	-4.557
ASN A 32	7.609	10.425	1.628	1.160	-7.39	-183.17	0.0000 // E=	-169.739
HISB A 33	1.424	2.927	6.244	1.139	-27.15	-12.75	0.0000 // E=	-28.172
GLU A 34	2.196	13.250	2.991	0.573	-23.25	3.75	0.0000 // E=	-0.492
GLU A 35	4.123	9.260	5.353	0.499	-14.90	4.99	0.0000 // E=	9.324
VAL A 36	3.699	6.079	0.872	2.157	-31.80	-9.66	0.0000 // E=	-28.660
ASP A 37	1.499	7.505	7.366	0.271	-40.11	-8.94	0.0000 // E=	-32.412
ALA A 38	0.827	3.836	2.745	0.095	-22.27	-9.86	0.0000 // E=	-24.631
TYR A 39	1.873	4.857	1.868	3.397	-42.00	-33.59	0.0000 // E=	-63.585
PRO A 40	1.138	17.097	17.913	0.312	-27.46	-24.63	0.0000 // E=	-15.630
VAL A 41	2.195	6.025	3.054	1.912	-21.85	9.36	0.0000 // E=	0.691
TYR A 42	2.295	6.857	3.000	0.914	-48.12	-44.06	0.0000 // E=	-79.113
ARG A 43	5.466	14.331	10.209	1.590	-50.89	-258.21	0.0000 // E=	-277.507
ASP A 44	3.280	18.272	10.055	0.990	-18.71	30.97	0.0000 // E=	44.854
PRO A 45	1.549	29.379	19.637	2.552	-11.66	-24.99	0.0000 // E=	16.465
VAL A 46	3.730	5.946	5.221	2.749	-20.20	7.70	0.0000 // E=	5.148
HISB A 47	1.559	5.287	3.510	3.224	-25.70	-26.82	0.0000 // E=	-38.945
ASP A 48	0.271	4.450	16.441	1.158	-16.06	-8.66	0.0000 // E=	-2.410
PHE A 49	1.998	3.840	21.851	1.998	-41.47	30.41	0.0000 // E=	18.627
GLY A 50	0.555	2.086	3.317	0.038	-26.97	27.21	0.0000 // E=	6.234
ILE A 51	0.798	2.719	1.730	0.783	-37.39	-11.40	0.0000 // E=	-42.760
LEU A 52	0.904	5.547	9.779	0.647	-47.27	-7.07	0.0000 // E=	-37.464
LYSH A 53	1.701	9.071	4.027	0.794	-24.99	-9.28	0.0000 // E=	-18.674
PHE A 54	1.939	5.244	5.838	0.389	-53.03	12.66	0.0000 // E=	-26.953
ASP A 55	2.206	17.798	5.335	0.634	3.46	31.20	0.0000 // E=	60.636
PRO A 56	0.553	13.868	18.926	3.898	-19.85	-22.29	0.0000 // E=	-4.894
LYSH A 57	4.127	39.709	15.776	6.422	14.58	0.45	0.0000 // E=	81.063
ALA A 58	6.479	22.474	2.419	7.790	-1.73	-5.15	0.0000 // E=	32.279
ILE A 59	2.684	24.885	44.580	11.729	-7.46	-6.32	0.0000 // E=	70.102
LYSH A 60	0.809	1.731	11.299	1.035	-30.06	-1.11	0.0000 // E=	-16.295
TYR A 61	1.682	7.391	6.460	0.719	-43.83	-41.99	0.0000 // E=	-69.572
MET A 62	0.460	7.364	4.802	0.478	-34.20	19.46	0.0000 // E=	-1.638
PRO A 63	0.268	17.310	21.521	0.872	-27.25	-26.58	0.0000 // E=	-13.860
VAL A 64	0.421	3.250	2.103	1.725	-11.91	1.13	0.0000 // E=	-3.284
ALA A 65	4.622	4.801	1.843	1.139	6.82	-0.92	0.0000 // E=	18.297
ALA A 66	0.378	2.325	1.955	0.047	-15.52	-13.32	0.0000 // E=	-24.130
LEU A 67	0.452	3.466	1.871	0.501	-11.51	30.22	0.0000 // E=	25.008
PRO A 68	0.564	16.525	18.827	2.356	-21.60	-27.38	0.0000 // E=	-10.710
LEU A 69	3.697	48.893	17.147	6.031	-2.17	6.03	0.0000 // E=	79.632
ARG A 70	129.422	197.265	45.997	38.112	105.41	-251.68	0.0000 // E=	264.522
PRO A 71	0.420	25.935	18.628	3.139	-24.89	-23.27	0.0000 // E=	-0.034
ASP A 72	0.838	4.859	4.875	0.175	-17.42	3.81	0.0000 // E=	-2.855
LEU A 73	0.901	7.126	4.918	0.294	-22.13	7.72	0.0000 // E=	-1.177
ALA A 74	0.516	0.883	4.542	0.376	-17.39	0.69	0.0000 // E=	-10.383
ARG A 75	1.813	7.030	15.223	1.023	-32.50	-259.43	0.0000 // E=	-266.837
VAL A 76	2.685	10.031	0.813	2.622	-2.02	35.52	0.0000 // E=	49.651
GLY A 77	9.732	83.498	3.827	40.014	14.87	27.47	0.0000 // E=	179.415
ILE A 78	12.730	39.280	4.050	29.248	-7.26	-20.94	0.0000 // E=	57.108
GLU A 79	2.709	8.190	9.885	0.437	-24.21	-1.14	0.0000 // E=	-4.131
ILE A 80	3.502	5.645	7.259	2.641	-35.05	-8.96	0.0000 // E=	-24.967
ARG A 81	5.448	22.257	29.663	24.935	0.59	-258.94	0.0000 // E=	-176.046
VAL A 82	2.681	6.371	11.619	2.316	-32.44	-9.75	0.0000 // E=	-19.201
VAL A 83	2.877	6.642	2.348	3.832	-29.96	27.52	0.0000 // E=	13.260
GLY A 84	1.050	0.854	6.334	0.208	-20.31	29.58	0.0000 // E=	17.718
ASN A 85	1.465	4.774	3.492	0.635	-29.42	-172.94	0.0000 // E=	-191.991
ASP A 86	1.156	5.704	2.858	0.422	-23.47	-3.17	0.0000 // E=	-16.497
ALA A 87	2.193	3.080	1.389	0.286	-15.03	38.02	0.0000 // E=	29.936
GLY A 88	1.993	5.247	2.680	0.116	-24.96	29.12	0.0000 // E=	14.195
GLU A 89	2.520	7.703	6.232	0.142	-16.02	-5.41	0.0000 // E=	-4.830
LYSH A 90	1.560	25.965	28.723	0.131	-36.17	-4.55	0.0000 // E=	15.664
LEU A 91	1.545	6.578	9.978	0.629	-33.48	-5.54	0.0000 // E=	-20.282
SER A 92	1.875	5.102	4.108	1.831	-24.41	-26.04	0.0000 // E=	-37.537
ILE A 93	3.442	5.205	0.453	1.734	-15.17	1.44	0.0000 // E=	-2.887
LEU A 94	2.089	5.656	3.101	1.080	-35.65	-12.80	0.0000 // E=	-36.523
SER A 95	2.542	3.173	3.421	0.790	-17.00	26.51	0.0000 // E=	19.436
GLY A 96	1.811	2.711	1.638	0.135	-18.85	28.26	0.0000 // E=	15.703
VAL A 97	2.441	4.861	1.840	2.335	-28.93	-13.15	0.0000 // E=	-30.602
ILE A 98	1.466	2.207	4.285	0.352	-40.33	-6.65	0.0000 // E=	-38.667
SER A 99	1.693	6.144	2.802	0.182	-8.65	-6.29	0.0000 // E=	-4.127
ARG A 100	2.120	3.282	8.489	0.596	-2.20	-259.87	0.0000 // E=	-247.584
LEU A 101	0.704	2.192	7.774	0.940	-5.15	3.77	0.0000 // E=	10.227
ASP A 102	1.212	4.643	7.802	0.717	-12.87	-1.68	0.0000 // E=	-0.171
ARG A 103	6.764	11.285	26.081	5.346	18.16	-262.01	0.0000 // E=	-194.376
ASN A 104	1.209	4.703	10.042	0.086	-2.65	-183.97	0.0000 // E=	-170.574
ALA A 105	0.224	2.830	1.249	0.089	-6.08	14.05	0.0000 // E=	12.363
PRO A 106	0.687	16.578	22.280	1.190	-14.89	-19.08	0.0000 // E=	6.767
GLU A 107	1.400	10.901	9.486	2.438	-40.44	4.01	0.0000 // E=	-12.207
TYR A 108	2.608	5.606	7.519	1.940	-35.69	1.29	0.0000 // E=	-16.728
GLY A 109	0.408	4.394	5.672	0.051	-7.53	44.64	0.0000 // E=	47.634
ASP A 110	0.622	2.228	4.081	1.762	-16.61	48.00	0.0000 // E=	40.086
GLY A 111	0.292	0.749	3.833	0.368	-6.71	43.85	0.0000 // E=	42.385
TYR A 112	2.098	24.508	5.255	0.883	-19.24	-43.12	0.0000 // E=	-29.620
SER A 113	1.634	161.819	9.548	7.054	-10.64	-30.62	0.0000 // E=	138.791
ASP A 114	1.663	9.251	6.176	2.488	-19.25	9.04	0.0000 // E=	9.365
PHE A 115	0.568	2.842	13.146	0.916	-24.23	16.08	0.0000 // E=	9.325
ASN A 116	4.273	21.892	5.235	4.064	-8.58	-165.41	0.0000 // E=	-138.520
THR A 117	2.078	3.372	3.603	1.706	-18.08	-20.47	0.0000 // E=	-27.783
CYSH A 118	3.278	19.082	3.801	2.027	-17.99	2.40	0.0000 // E=	12.598
TYR A 119	4.503	17.722	3.907	3.159	-41.96	-57.47	0.0000 // E=	-70.133

TYR	A	120	3.469	29.658	7.084	17.527	-52.19	-43.14	0.0000	// E=	-37.594
GLN	A	121	2.141	11.624	4.288	14.672	-33.53	-166.01	0.0000	// E=	-166.815
ALA	A	122	0.789	2.647	0.358	0.084	-21.32	-3.90	0.0000	// E=	-21.336
SER	A	123	2.424	3.790	4.872	0.426	-14.95	-18.57	0.0000	// E=	-22.008
ALA	A	124	1.668	3.981	3.294	0.026	-13.36	-8.08	0.0000	// E=	-12.474
ALA	A	125	0.978	2.677	2.939	0.889	-10.48	0.09	0.0000	// E=	-2.901
ALA	A	126	1.214	1.335	4.065	0.445	-16.19	-4.63	0.0000	// E=	-13.761
SER	A	127	1.841	3.573	2.801	0.977	-19.19	17.61	0.0000	// E=	7.616
GLY	A	128	1.096	1.440	0.740	0.841	-8.52	74.75	0.0000	// E=	70.343
GLY	A	129	0.810	7.580	2.943	0.356	-10.00	32.57	0.0000	// E=	34.256
SER	A	130	2.139	3.251	8.597	1.057	-28.43	-22.74	0.0000	// E=	-36.128
SER	A	131	1.517	6.167	3.090	0.723	-10.33	36.54	0.0000	// E=	37.698
GLY	A	132	0.390	8.780	1.612	0.026	-18.39	40.53	0.0000	// E=	32.946
SER	A	133	3.137	7.178	15.136	0.140	-21.30	9.08	0.0000	// E=	13.379
PRO	A	134	14.174	56.754	8.696	3.911	33.67	-28.90	0.0000	// E=	88.309
VAL	A	135	2.116	4.553	9.345	0.167	-27.42	3.97	0.0000	// E=	-7.264
VAL	A	136	82.716	103.096	3.095	13.546	155.70	-18.54	0.0000	// E=	339.608
ASN	A	137	20.026	4.533	5.266	0.982	-14.04	-150.70	0.0000	// E=	-133.933
LYSH	A	138	5.166	36.285	15.670	0.724	17.72	1.74	0.0000	// E=	77.308
ASP	A	139	0.374	2.938	15.779	0.334	-16.91	47.86	0.0000	// E=	50.379
GLY	A	140	0.769	0.527	0.598	0.088	-12.80	46.35	0.0000	// E=	35.531
PHE	A	141	18.218	33.459	7.372	14.247	19.91	2.31	0.0000	// E=	95.516
ALA	A	142	0.766	1.132	1.965	0.594	-19.92	-8.87	0.0000	// E=	-24.337
VAL	A	143	26.803	53.610	4.298	2.344	87.01	-2.81	0.0000	// E=	171.256
ALA	A	144	3.913	5.589	0.614	1.041	-9.98	2.23	0.0000	// E=	3.411
LEU	A	145	1.527	9.781	9.456	0.849	-46.43	5.84	0.0000	// E=	-18.982
GLN	A	146	9.962	58.995	5.616	25.445	-26.53	-184.45	0.0000	// E=	-110.961
ALA	A	147	2.183	15.903	5.336	16.918	-8.54	50.57	0.0000	// E=	82.378
GLY	A	148	0.838	1.902	7.567	1.227	-5.88	91.45	0.0000	// E=	97.109
GLY	A	149	0.471	2.874	2.440	0.190	-13.27	54.00	0.0000	// E=	46.701
ARG	A	150	1.664	4.488	8.298	2.545	-20.63	-261.49	0.0000	// E=	-265.121
ALA	A	151	0.200	1.231	5.133	0.261	-18.06	-1.14	0.0000	// E=	-12.382
ASP	A	152	0.311	5.790	12.891	0.599	-12.57	44.22	0.0000	// E=	51.240
GLY	A	153	0.157	3.140	7.036	0.047	-9.67	46.16	0.0000	// E=	46.867
ALA	A	154	0.428	2.270	27.995	2.020	-10.72	8.13	0.0000	// E=	30.120
SER	A	155	1.559	12.128	27.903	1.588	1.47	49.30	0.0000	// E=	93.950
//------------											
KJ/mol			592.644	2001.879	1315.600	453.844	-2700.92	-2962.41	0.0000	// E=	-1299.371

// Computations were done in vacuo with the GROMOS96 43B1 parameters set, without reaction field.
// For more information about GROMOS96, refer to: W.F. van Gunsteren et al. (1996) in Biomolecular
// simulation: the GROMOS96 manual and user guide. Vdf Hochschulverlag ETHZ (http://igc.ethz.ch/gromos).
// When using those results, please mention that energy computations were done with the GROMOS96
// implementation of Swiss-PdbViewer.

Table S4. Energy parameters of modeled PA protease structure from *Arabidopsis thaliana*

residue	bonds	angles	torsion	improper	nonBonded	electrostatic	constraint //	TOTAL
GLY A 1	0.528	0.749	0.381	0.627	-5.30	161.25	0.0000 // E=	158.236
SER A 2	2.167	7.999	7.124	1.571	-14.40	31.40	0.0000 // E=	35.866
GLY A 3	0.776	2.477	2.951	0.334	-21.10	35.86	0.0000 // E=	21.293
VAL A 4	3.005	4.761	2.679	1.616	-38.14	-12.33	0.0000 // E=	-38.411
VAL A 5	3.223	3.015	4.639	1.903	-40.78	-9.47	0.0000 // E=	-37.472
TRP A 6	2.476	14.741	2.266	4.904	-37.86	-16.57	0.0000 // E=	-30.048
ASP A 7	1.400	8.852	1.380	2.175	-24.24	40.94	0.0000 // E=	30.501
GLY A 8	0.639	2.146	3.602	0.095	-12.89	43.85	0.0000 // E=	37.451
GLN A 9	2.312	5.803	3.919	1.468	-14.95	-115.34	0.0000 // E=	-116.785
GLY A 10	0.499	14.044	7.509	0.056	-21.51	32.10	0.0000 // E=	32.701
TYR A 11	1.093	3.631	7.084	1.525	-47.75	-46.61	0.0000 // E=	-81.024
ILE A 12	2.366	12.739	6.074	2.133	-31.69	-10.90	0.0000 // E=	-19.279
VAL A 13	2.962	3.513	2.251	0.947	-33.78	-10.03	0.0000 // E=	-34.131
THR A 14	2.695	9.485	6.028	0.927	-25.28	-27.43	0.0000 // E=	-33.581
ASN A 15	1.896	6.497	4.226	1.714	-42.01	-157.78	0.0000 // E=	-185.453
TYR A 16	1.028	3.528	4.885	1.391	-63.88	-52.56	0.0000 // E=	-105.614
HISB A 17	0.520	5.206	3.057	2.123	-28.67	3.23	0.0000 // E=	-14.531
VAL A 18	1.601	6.790	0.868	5.394	-22.97	5.75	0.0000 // E=	-2.568
ILE A 19	3.231	4.886	5.054	2.805	-26.26	31.93	0.0000 // E=	21.649
GLY A 20	0.847	6.065	2.571	1.189	-14.64	32.40	0.0000 // E=	28.430
ASN A 21	0.894	7.013	9.934	0.596	-21.79	-172.61	0.0000 // E=	-175.968
ALA A 22	6.016	3.083	3.733	0.329	-5.27	-7.82	0.0000 // E=	0.067
LEU A 23	1.908	19.188	8.224	1.043	-20.25	-0.87	0.0000 // E=	9.246
SER A 24	0.546	18.366	47.938	0.504	-20.81	-2.57	0.0000 // E=	43.973
ARG A 25	1.743	7.156	11.437	6.130	-20.26	-267.82	0.0000 // E=	-261.619
ASN A 26	2.147	11.458	3.971	0.423	-21.54	-136.10	0.0000 // E=	-139.640
PRO A 27	0.647	13.546	31.688	2.735	-27.31	-24.54	0.0000 // E=	-3.230
SER A 28	5.124	10.650	4.617	2.820	-13.90	13.38	0.0000 // E=	22.692
PRO A 29	11.265	80.629	29.955	22.947	-23.39	7.35	0.0000 // E=	128.761
GLY A 30	1.840	11.466	35.831	14.056	-12.01	41.35	0.0000 // E=	92.539
ASP A 31	1.108	4.608	4.303	1.607	-15.87	-10.23	0.0000 // E=	-14.475
VAL A 32	0.919	5.366	1.343	1.733	-21.07	7.80	0.0000 // E=	-3.909
VAL A 33	1.549	6.602	13.326	4.175	-15.06	39.17	0.0000 // E=	49.766
GLY A 34	1.493	5.364	4.474	1.523	-22.38	21.94	0.0000 // E=	12.421
ARG A 35	4.417	13.326	22.140	2.666	-45.55	-281.52	0.0000 // E=	-284.526
VAL A 36	1.297	3.543	12.525	2.219	-22.69	6.01	0.0000 // E=	2.904
ASN A 37	1.787	11.570	4.820	2.333	-3.71	-179.48	0.0000 // E=	-162.676
ILE A 38	0.873	5.309	2.437	0.422	1.94	-0.15	0.0000 // E=	10.826
LEU A 39	1.868	38.137	4.921	3.641	-25.20	-6.66	0.0000 // E=	16.710
ALA A 40	2.683	176.735	57.451	1.989	-23.28	-11.40	0.0000 // E=	204.181
SER A 41	0.611	9.469	17.570	1.941	-12.45	-3.31	0.0000 // E=	13.837
ASP A 42	0.699	7.628	5.799	1.513	-29.76	54.11	0.0000 // E=	39.992
GLY A 43	0.643	0.409	5.372	0.491	-13.26	41.23	0.0000 // E=	34.880
VAL A 44	2.123	8.438	6.455	6.528	-20.34	11.63	0.0000 // E=	14.831
GLN A 45	2.044	26.701	5.935	0.922	-35.82	-178.22	0.0000 // E=	-178.438
LYSH A 46	2.358	28.819	17.019	9.876	-17.11	0.83	0.0000 // E=	41.785
ASN A 47	1.377	6.612	5.726	2.811	-24.08	-166.99	0.0000 // E=	-174.544
PHE A 48	0.819	14.108	3.242	3.870	-34.95	-1.03	0.0000 // E=	-13.938
GLU A 49	1.342	15.978	15.218	5.969	9.65	29.26	0.0000 // E=	77.425
GLY A 50	0.791	2.189	2.400	0.322	-12.82	40.32	0.0000 // E=	33.198
LYSH A 51	1.476	11.996	4.371	1.713	-23.42	-3.63	0.0000 // E=	-7.499
LEU A 52	0.665	6.876	6.566	1.673	-41.07	1.58	0.0000 // E=	-23.711
VAL A 53	2.931	19.171	32.823	10.795	-12.44	44.59	0.0000 // E=	97.868
GLY A 54	0.769	6.029	3.375	6.065	-17.19	26.18	0.0000 // E=	25.228
ALA A 55	0.566	1.645	9.687	0.685	-16.49	-4.46	0.0000 // E=	-8.364
ASP A 56	0.641	2.173	1.570	1.164	-26.11	-14.73	0.0000 // E=	-35.288
ARG A 57	2.113	10.115	6.133	4.190	-30.49	-262.15	0.0000 // E=	-270.087
ALA A 58	0.854	2.288	7.179	2.643	-14.56	-2.85	0.0000 // E=	-4.445
LYSH A 59	2.498	7.701	2.116	0.724	-23.69	8.36	0.0000 // E=	-2.300
ASP A 60	2.164	5.382	11.643	2.566	-32.37	-8.95	0.0000 // E=	-19.566
LEU A 61	1.180	8.401	2.270	0.332	-47.95	-9.26	0.0000 // E=	-45.023
ALA A 62	1.023	0.911	18.775	0.709	-34.48	-8.33	0.0000 // E=	-21.393
VAL A 63	2.383	3.516	3.851	1.436	-35.33	-15.29	0.0000 // E=	-39.437
LEU A 64	2.058	13.714	2.153	0.581	-53.82	-13.77	0.0000 // E=	-49.089
LYSH A 65	1.521	8.051	29.330	3.780	-41.94	-1.32	0.0000 // E=	-0.572
VAL A 66	0.946	1.808	7.343	1.371	-31.38	-5.59	0.0000 // E=	-25.506
ASP A 67	2.599	8.779	5.111	1.310	-2.24	-1.26	0.0000 // E=	14.295
ALA A 68	0.470	1.719	2.379	0.254	-17.65	16.70	0.0000 // E=	3.869
PRO A 69	0.524	16.040	17.143	0.246	-13.49	-23.00	0.0000 // E=	-2.541
GLU A 70	1.341	4.909	3.959	1.791	-10.07	-0.57	0.0000 // E=	1.360
THR A 71	1.679	7.802	5.534	4.004	-4.29	0.00	0.0000 // E=	14.729
LEU A 72	1.407	6.006	2.755	1.115	-14.96	14.04	0.0000 // E=	10.367
LEU A 73	0.635	3.221	3.251	0.632	-35.08	6.82	0.0000 // E=	-20.522
LYSH A 74	0.802	11.702	17.645	0.456	-35.23	28.19	0.0000 // E=	23.569
PRO A 75	0.694	15.398	17.157	0.683	-25.05	-31.40	0.0000 // E=	-22.513
ILE A 76	3.421	7.368	5.256	2.270	-3.57	4.40	0.0000 // E=	19.149
LYSH A 77	6.205	49.216	9.234	7.326	-12.58	4.37	0.0000 // E=	63.777
VAL A 78	3.805	19.730	5.316	19.932	-16.01	46.03	0.0000 // E=	78.799
GLY A 79	0.616	0.929	3.416	0.127	-10.54	37.10	0.0000 // E=	31.647
GLN A 80	1.929	7.168	5.038	0.993	-14.69	-158.26	0.0000 // E=	-157.820
SER A 81	1.789	4.290	3.656	1.010	-10.18	-8.44	0.0000 // E=	-7.880
ASN A 82	2.580	7.799	8.973	1.377	-27.78	-173.26	0.0000 // E=	-180.307
SER A 83	0.857	4.821	11.306	0.554	-14.25	-12.57	0.0000 // E=	-9.283
LEU A 84	1.580	6.211	6.607	0.927	-29.79	1.74	0.0000 // E=	-12.724
LYSH A 85	2.490	8.716	13.143	0.214	-31.80	-4.28	0.0000 // E=	-11.517
VAL A 86	2.601	2.227	5.178	1.669	-18.19	37.34	0.0000 // E=	30.818
GLY A 87	0.910	8.753	3.753	0.134	-11.96	28.72	0.0000 // E=	30.312
GLN A 88	3.413	11.033	7.285	0.590	-36.74	-179.86	0.0000 // E=	-194.272
GLN A 89	2.732	4.566	4.342	0.693	-26.80	-164.08	0.0000 // E=	-178.544
CYSH A 90	1.006	3.301	9.753	0.047	-36.71	-19.21	0.0000 // E=	-41.810
LEU A 91	0.623	2.617	9.555	0.527	-37.68	-10.48	0.0000 // E=	-34.846
ALA A 92	0.401	1.318	4.233	0.404	-22.59	-11.67	0.0000 // E=	-27.896
ILE A 93	1.059	4.091	7.934	2.024	-44.89	31.47	0.0000 // E=	1.695
GLY A 94	1.426	11.591	6.692	1.342	-14.10	33.72	0.0000 // E=	40.671
ASN A 95	2.271	18.781	68.412	6.438	-32.80	-137.00	0.0000 // E=	-73.901
PRO A 96	0.424	19.443	23.057	0.556	-30.65	-25.83	0.0000 // E=	-12.998
PHE A 97	0.854	3.194	8.340	2.809	-22.34	51.80	0.0000 // E=	44.655
GLY A 98	0.365	0.622	4.343	0.111	-9.29	49.08	0.0000 // E=	45.231
PHE A 99	0.245	1.498	9.916	0.217	-18.66	7.93	0.0000 // E=	1.145
ASP A 100	0.320	11.236	4.728	2.747	-29.60	8.56	0.0000 // E=	-2.006
HISA A 101	0.925	2.511	6.110	2.691	-35.63	5.47	0.0000 // E=	-17.922
THR A 102	1.728	4.760	2.228	2.140	-36.09	-12.67	0.0000 // E=	-37.907
LEU A 103	2.368	6.558	6.070	1.313	-15.91	0.85	0.0000 // E=	1.251
THR A 104	3.174	6.328	5.343	1.916	-27.54	-14.91	0.0000 // E=	-25.688
VAL A 105	3.096	4.370	2.143	1.809	-20.16	33.76	0.0000 // E=	25.023
GLY A 106	1.811	4.136	4.196	0.742	-20.98	19.41	0.0000 // E=	9.313
VAL A 107	2.490	3.472	1.598	1.142	-24.34	-7.26	0.0000 // E=	-22.899
ILE A 108	4.133	5.842	3.226	1.292	-33.67	-11.58	0.0000 // E=	-30.755
SER A 109	1.968	4.218	3.888	1.878	-22.97	38.29	0.0000 // E=	27.275
GLY A 110	1.628	2.119	7.392	0.907	-19.77	43.87	0.0000 // E=	36.146
LEU A 111	1.864	3.252	4.455	1.407	-31.60	-0.50	0.0000 // E=	-21.127
ASN A 112	1.422	7.418	3.041	1.047	-33.02	-160.37	0.0000 // E=	-180.462
ARG A 113	1.516	4.407	6.672	0.272	-23.42	-253.51	0.0000 // E=	-264.057
ASP A 114	0.483	3.242	11.457	3.934	-17.70	6.13	0.0000 // E=	7.548
ILE A 115	2.927	7.264	3.888	1.622	-17.68	-4.85	0.0000 // E=	-6.834
PHE A 116	0.776	3.796	4.951	2.380	-27.18	2.43	0.0000 // E=	-12.845
SER A 117	1.016	6.429	9.978	0.251	-14.13	-11.79	0.0000 // E=	-8.243
GLN A 118	1.308	6.184	8.747	1.912	-35.19	-155.41	0.0000 // E=	-172.448

THR A 119	1.234	4.416	4.295	1.871	-10.01	17.12	0.0000	// E=	18.925
GLY A 120	0.797	0.761	11.439	0.217	-16.71	26.09	0.0000	// E=	22.593
VAL A 121	1.323	4.840	4.536	2.677	-19.41	-8.70	0.0000	// E=	-14.733
THR A 122	2.043	4.078	3.294	2.587	-13.14	-6.16	0.0000	// E=	-7.291
ILE A 123	3.609	6.516	4.042	1.982	-9.49	46.00	0.0000	// E=	52.653
GLY A 124	0.901	2.469	0.333	0.522	-20.36	70.67	0.0000	// E=	54.533
GLY A 125	0.796	9.857	3.525	1.022	-17.58	72.61	0.0000	// E=	70.229
GLY A 126	0.431	3.868	1.406	0.528	-17.47	28.56	0.0000	// E=	17.329
ILE A 127	2.742	5.541	11.505	1.898	-33.01	-9.26	0.0000	// E=	-20.584
GLN A 128	2.305	5.672	5.253	0.873	-32.41	-173.62	0.0000	// E=	-191.921
THR A 129	2.090	6.129	1.741	2.006	-36.54	-21.96	0.0000	// E=	-46.530
ASP A 130	1.190	4.546	8.472	0.677	-17.09	5.55	0.0000	// E=	3.347
ALA A 131	0.357	1.354	3.376	0.024	-22.73	2.23	0.0000	// E=	-15.397
ALA A 132	0.788	1.485	1.080	0.428	-15.25	-9.31	0.0000	// E=	-20.779
ILE A 133	2.408	3.583	3.438	2.753	-35.17	-2.35	0.0000	// E=	-25.337
ASN A 134	2.099	6.562	2.949	1.714	-29.39	-160.06	0.0000	// E=	-176.118
PRO A 135	0.682	19.326	25.241	0.798	-8.64	14.17	0.0000	// E=	51.577
GLY A 136	0.476	1.166	1.384	0.095	-17.17	44.77	0.0000	// E=	30.720
ASN A 137	1.716	6.167	5.208	0.678	-39.86	-162.02	0.0000	// E=	-188.108
SER A 138	1.954	7.014	5.441	0.512	-28.44	16.57	0.0000	// E=	3.049
GLY A 139	1.310	8.209	0.229	1.013	-23.38	70.34	0.0000	// E=	57.721
GLY A 140	1.899	6.554	2.283	0.077	-19.91	44.02	0.0000	// E=	34.923
PRO A 141	1.637	20.334	18.042	0.355	-41.05	-27.13	0.0000	// E=	-27.805
LEU A 142	2.124	10.805	4.048	0.762	-46.16	-3.76	0.0000	// E=	-32.180
LEU A 143	1.339	12.023	5.476	0.300	-46.33	-14.11	0.0000	// E=	-41.303
ASP A 144	1.768	4.373	10.505	4.522	-31.33	11.25	0.0000	// E=	1.080
SER A 145	0.784	7.705	1.337	2.090	-9.87	-10.40	0.0000	// E=	-8.357
LYSH A 146	1.439	17.121	6.329	0.371	-18.29	46.20	0.0000	// E=	53.177
GLY A 147	0.883	2.723	9.576	0.732	-19.36	48.66	0.0000	// E=	43.211
ASN A 148	2.913	6.295	7.454	0.994	-38.09	-168.49	0.0000	// E=	-188.917
LEU A 149	1.384	7.805	3.671	0.995	-33.57	-5.28	0.0000	// E=	-24.997
ILE A 150	1.791	8.368	34.776	4.396	-30.37	38.11	0.0000	// E=	57.070
GLY A 151	0.381	3.970	0.335	1.028	-16.97	40.34	0.0000	// E=	29.086
ILE A 152	2.221	7.982	16.448	0.672	-25.37	-3.65	0.0000	// E=	-1.694
ASN A 153	2.566	4.050	14.660	0.237	-50.72	-187.55	0.0000	// E=	-216.758
THR A 154	3.161	5.912	0.861	0.830	-37.48	-14.62	0.0000	// E=	-41.328
ALA A 155	0.905	1.495	0.502	0.403	-25.03	-3.92	0.0000	// E=	-25.646
ILE A 156	1.701	3.582	2.258	1.866	-26.11	-5.59	0.0000	// E=	-22.296
PHE A 157	0.509	4.950	6.867	0.634	-25.93	5.59	0.0000	// E=	-7.383
THR A 158	2.289	3.277	26.916	1.812	-15.97	-18.59	0.0000	// E=	-0.269
GLN A 159	4.351	56.755	6.627	10.858	-7.32	-160.22	0.0000	// E=	-88.955
THR A 160	0.786	49.027	28.239	13.191	5.60	32.19	0.0000	// E=	129.034
GLY A 161	0.649	2.128	2.794	0.061	-12.92	31.43	0.0000	// E=	24.138
THR A 162	0.301	1.217	3.859	0.609	-16.82	-10.27	0.0000	// E=	-21.110
SER A 163	0.315	4.459	4.160	1.106	-13.99	-4.04	0.0000	// E=	-7.992
ALA A 164	0.518	0.690	13.541	0.728	-11.32	30.28	0.0000	// E=	34.433
GLY A 165	0.281	1.244	3.968	0.010	-13.10	40.67	0.0000	// E=	33.077
VAL A 166	0.458	1.459	0.349	1.226	-20.90	27.94	0.0000	// E=	10.528
GLY A 167	0.369	0.703	2.306	0.007	-16.74	20.65	0.0000	// E=	7.299
PHE A 168	0.570	1.709	5.223	0.513	-47.51	-5.42	0.0000	// E=	-44.921
ALA A 169	1.207	1.938	0.483	0.575	-36.04	-10.23	0.0000	// E=	-42.068
ILE A 170	5.109	7.620	3.105	1.928	-29.46	17.90	0.0000	// E=	6.203
PRO A 171	2.664	19.064	22.639	2.377	-22.81	31.53	0.0000	// E=	55.465
KJ/mol	291.422	1598.663	1432.665	358.775	-4068.57	-2896.47	0.0000	// E=	-3283.516

// Computations were done in vacuo with the GROMOS96 43B1 parameters set, without reaction field.
// For more information about GROMOS96, refer to: W.F. van Gunsteren et al. (1996) in Biomolecular
// simulation: the GROMOS96 manual and user guide. Vdf Hochschulverlag ETHZ (http://igc.ethz.ch/gromos).
// When using those results, please mention that energy computations were done with the GROMOS96
// implementation of Swiss-PdbViewer.

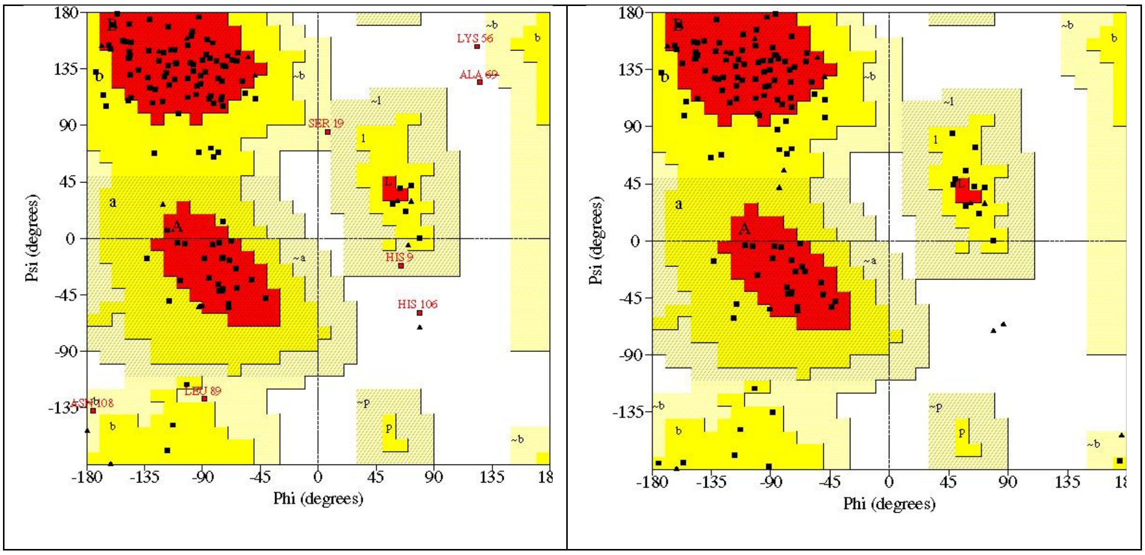

Figure S1. Ramachandran plot of φ-ψ dihedral angles of a modeled PA serine protease structure from *Plasmodium falciparum* before and after backbone refinement. PROCHECK was used to check the distribution of φ-ψ dihedral angles and eliminate Ramachandran outliers in the modeled protease structure (A, before; B, after refinement). Residues whose φ-ψ pairs fell outside the most favourable (red) and additional allowed (yellow) zones are annotated in red.

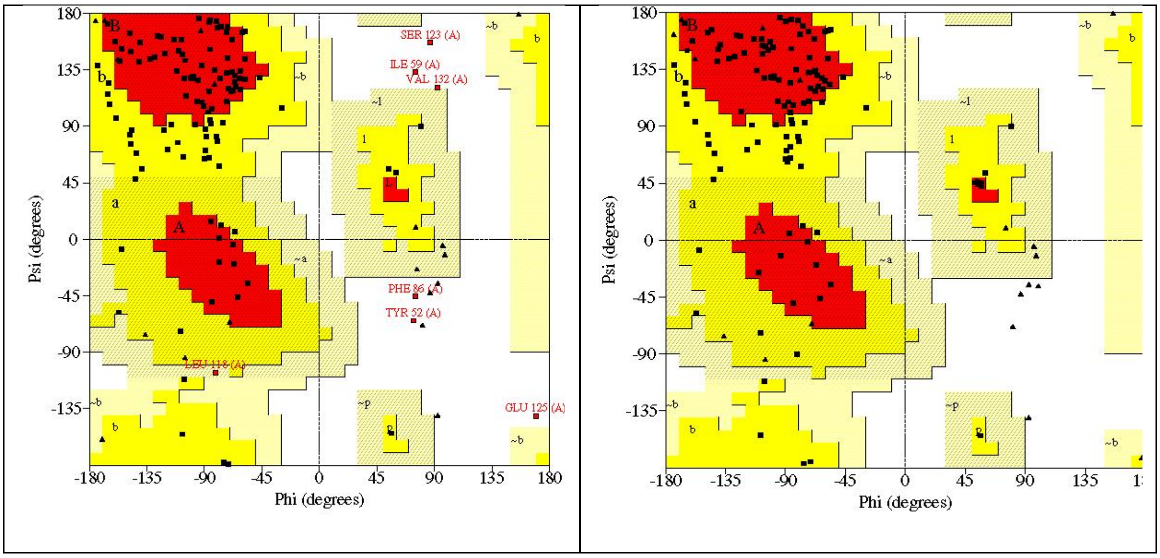

Figure S2. Ramachandran plot of φ-ψ dihedral angles of a modeled PA serine protease structure from *Pyrococcus furiosus* before and after backbone refinement. PROCHECK was used to check the distribution of φ-ψ dihedral angles and eliminate Ramachandran outliers in the modeled protease structure (A, before; B, after refinement). Residues whose φ-ψ pairs fell outside the most favourable (red) and additional allowed (yellow) zones are annotated in red.

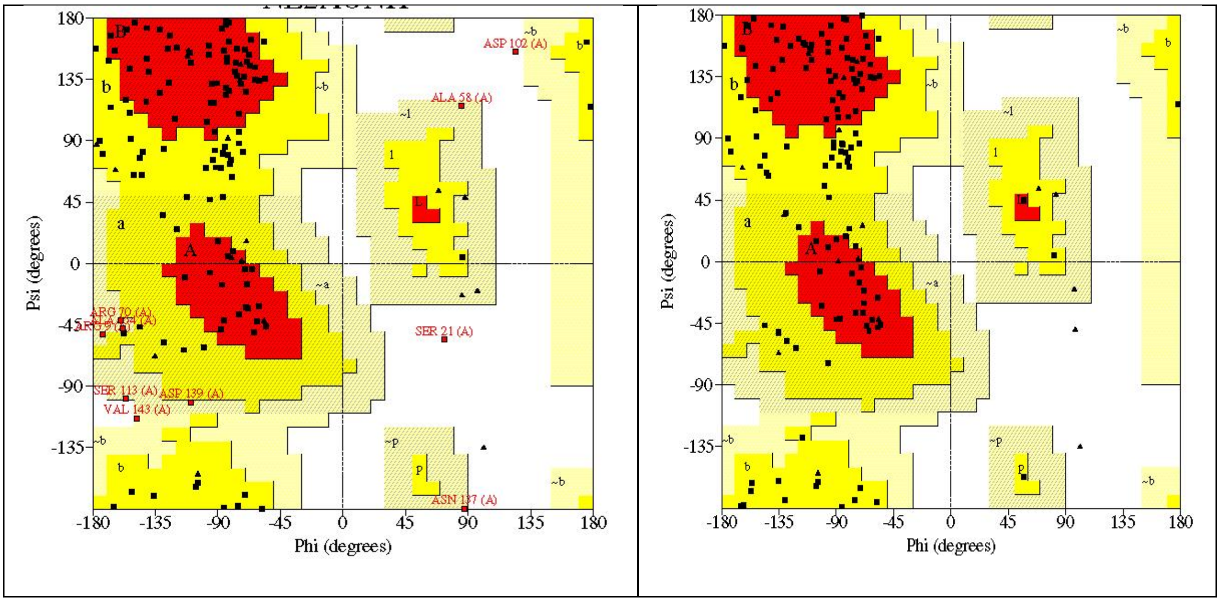

Figure S3. Ramachandran plot of φ-ψ dihedral angles of a modeled PA serine protease structure from *Neurospora crassa* before and after backbone refinement. PROCHECK was used to check the distribution of φ-ψ dihedral angles and eliminate Ramachandran outliers in the modeled protease structure (A, before; B, after refinement). Residues whose φ-ψ pairs fell outside the most favourable (red) and additional allowed (yellow) zones are annotated in red.

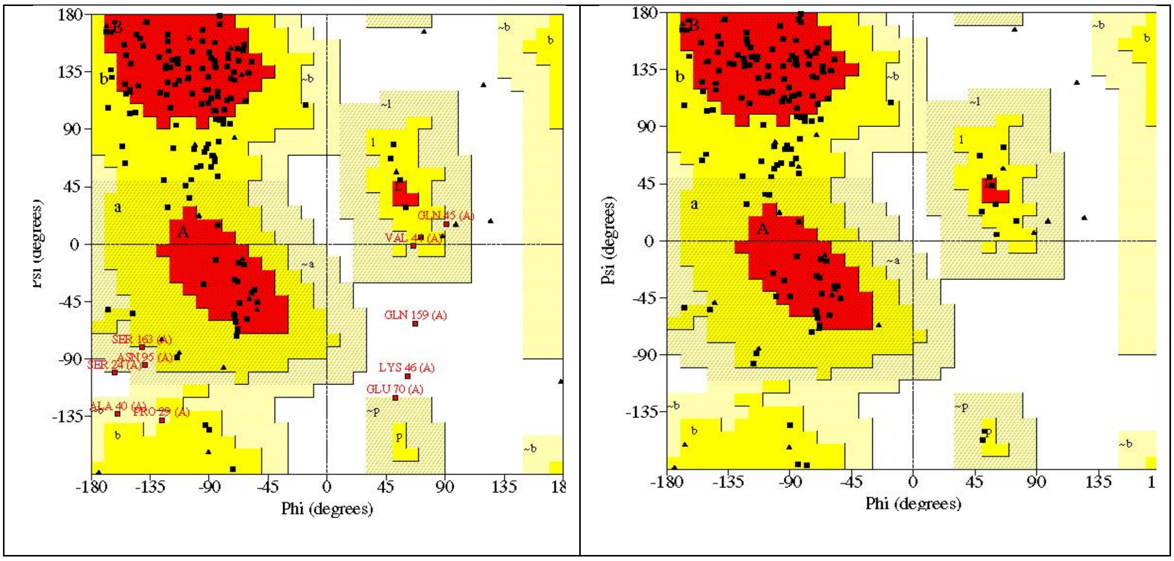

Figure S4. Ramachandran plot of φ-ψ dihedral angles of a modeled PA serine protease structure from *Arabidopsis thaliana* before and after backbone refinement. PROCHECK was used to check the distribution of φ-ψ dihedral angles and eliminate Ramachandran outliers in the modeled protease structure (A, before; B, after refinement). Residues whose φ-ψ pairs fell outside the most favourable (red) and additional allowed (yellow) zones are annotated in red.

Table S5. Predicted hydrogen bonds in modeled PA protease structures

B. taurus **(*CTRB*), for numbering reference**	***P. falciparum*** **(PM0075793)**	***P. furiosus*** **(PM0075794)**	***N. crassa*** **(PM0075795)**	***A. thaliana*** **(PM0075796)**
Thr54	Thr325<br>(Ser314, 2.69 Å)	Thr283<br>(Ala302, 2.90 Å)	Thr117<br>(Gly153, 2.83 Å)<br>(Asp189, 2.95 Å)	Thr96<br>(Ala144, 2.87 Å)
Ala56	Ala327<br>(Ser331, 2.88 Å)<br>(Asp359, 2.99 Å)			Ala98<br>(Asp130, 2.84 Å)
**His57**	**His328**	**His286<br>(Asp320, 2.87 Å)**	**His120<br>(Asp151, 2.45 Å)**	**His99**
**Asp102**	**Asp359<br>(Ala327, 2.99 Å)**	**Asp320<br>(His286, 2.87 Å)<br>(Gly285, 2.76 Å)<br>(Thr381, 2.92 Å)**	**Asp151<br>(His120, 2.45 Å)**	**Asp130<br>(Ala98, 2.84 Å)**
Gly193	Gly436<br>(Ser314, 2.95 Å)<br>(Asp433, 2.90 Å)	Gly387	Gly222	
Asp194		Asp388<br>(Arg360, 2.92 Å)<br>(Thr361, 2.67 Å)		
**Ser195**	**Ser438<br>(Ser314, 2.79 Å)<br>(Asp329, 2.70 Å)<br>(Asp433, 2.91 Å)**	**Ser389<br>(Thr268, 2.92 Å)**	**Ser234<br>(Gln249, 2.63 Å)<br>(Ser236, 2.81 Å)**	**Ser208<br>(Ser84, 2.63 Å)<br>(Asn216, 2.93 Å)**
Gly196	Gly439	Gly390	Gly235<br>(Gly2, 2.59 Å)	Gly209
Gly197	Gly440<br>(Asp436, 2.97 Å)			
Pro198	Pro441	Pro392	Pro237	

DeepView/Swiss-PbdViewer was used to calculate potential hydrogen bonds between residues of modeled PA protease structures with either a hydrogen molecule present (1.20-2.76 Å, minimum angle 120º) or not present (2.19-3.00 Å, minimum angle 90º). Highly conserved amino acids (including catalytic triad residues in bold) from the multiple sequence alignment are listed with potential binding partners, including the minimum bond length (Å). Bovine chymotrypsin B (*CTRB*) is included as a standard reference for residue numbering.

Table S6. Disulfide bonds in close proximity to catalytic histidine residue of experimental structures and modeled structures of PA serine proteases

PA protease structure	Disulfide bond pairs	Distance in amino acids between cysteine residues
1A0L	(C59 ↔ C75)	16
1JRS	(C48 ↔ C64)	16
1DPO	(C48 ↔ C64)	16
1BIT	(C45 ↔ C61)	16
1AO5	(C50 ↔ C66)	16
1BQY	(C50 ↔ C66)	16
1SGI	(C391 ↔ C407)	16
1ABJ	(C391 ↔ C407)	16
2ANY	(C419 ↔ C435)	16
1TRY	(C50 ↔ C66)	16
1EKB	(C826 ↔ C842)	16
**1M9U**	**(C29 ↔ C45) 2.03 Å**	**16**
1EQ9	(C26 ↔ C42)	16
1ARC	(C241 ↔ C263)	22
1QY6	-	-
1SGC	(C130 ↔ C150)	20
PM0075796	-	-
**PM0075794**	**(C267 ↔ C287) 2.04 Å**	**20**
PM0075793	-	-
PM0075795	-	-

For experimental PA serine protease structures, a disulfide bond in close proximity to the catalytic histidine residue has been previously described and is annotated in UniProt (with the exception of 1M9U). For the modeled PA serine proteases, only PM0075794 (*P. furiosus*) had a cysteine residue close to the catalytic histidine. Based on homology, we predict a disulfide bond between C29 and C45 (2.03 Å) of the M9U structure (*E. fetida*) and between C267 and C287 2.04 Å of the PM0075794 modeled structure (as shown in Figure S5).

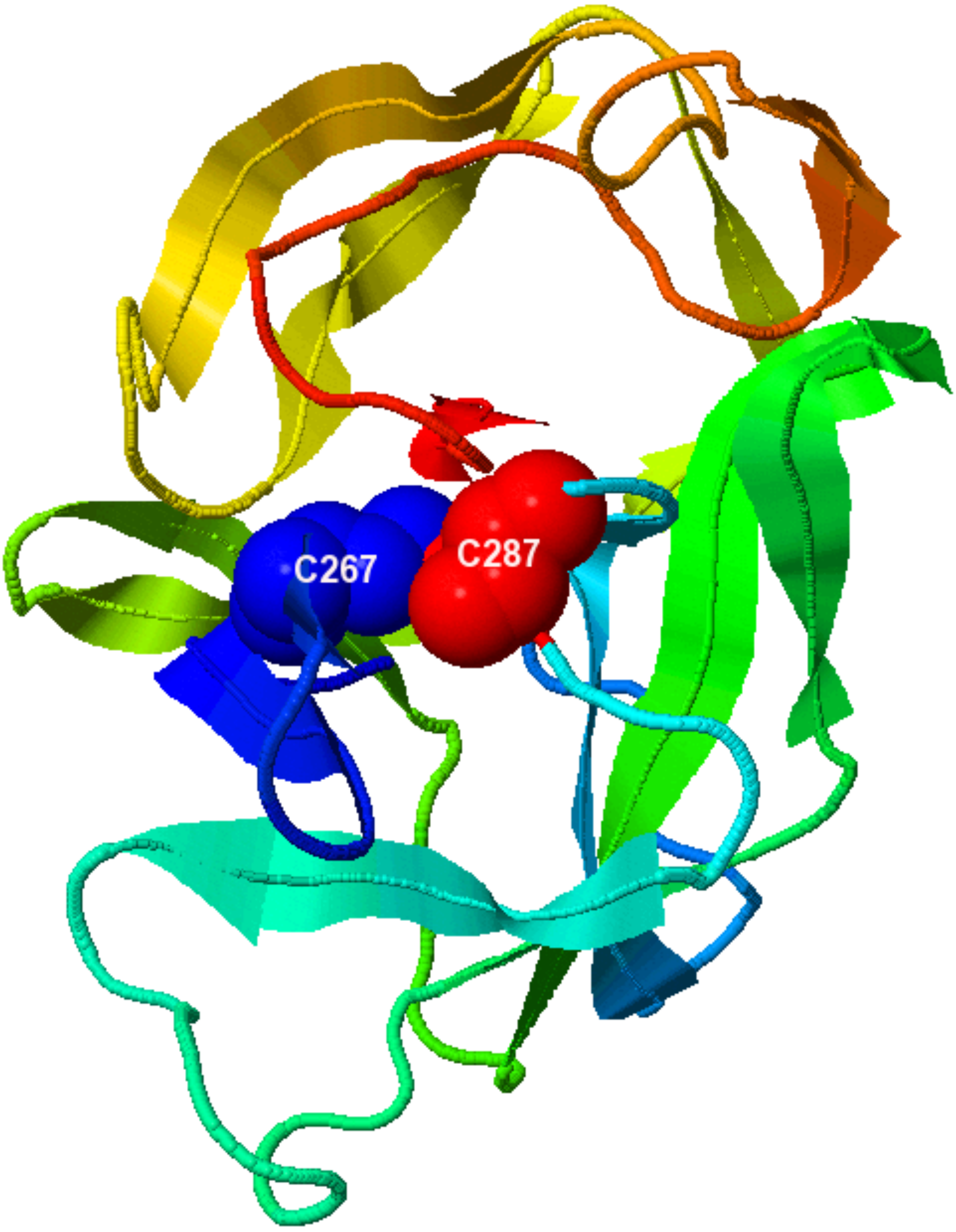

Figure S5. Predicted disulfide bond in Modeled PA protease structure of *Pyrococcus furiosus* (PMDB ID: PM0075794). The ribbon model shows secondary structures (β-sheets with arrow directed to C-terminus, α-helices and turn/loops) in alternating colors and cysteine residues Cys 267 (blue) and Cys287 (red) forming a predicted disulfide bond (2.04 Å).

Table S7. Relative comparison of PA serine protease amino acid composition based on physico-chemical properties

ID	Small (%)	Aliphatic (%)	Aromatic (%)	Nonpolar (%)	Polar (%)	Charged (%)	Basic (%)	Acidic (%)
**All residues**								
CTRB	43	24	12	45	55	31	19	12
1ARC-A	67	16	9	54	46	18	9	8
1QY6-A	63	16	9	46	54	22	10	12
1SGC-A	69	18	8	58	42	13	8	5
1TRY-A	72	20	7	61	39	10	6	4
1EKB-B	54	21	12	54	46	20	8	11
1JRS-A	63	21	9	57	43	14	9	6
1M9U-A	70	20	8	57	43	11	5	6
1SGI-B	52	18	11	52	48	28	14	14
1A0L-A	54	24	13	63	37	21	12	9
1ABJ-H	52	18	11	52	48	28	14	14
2ANY-A	52	19	13	53	47	21	13	8
1AO5-A	54	23	10	61	39	20	11	8
1DPO-A	61	24	9	61	39	17	8	10
1BIT-A	61	20	12	58	42	15	7	8
1EQ9-A	60	25	9	53	47	23	12	11
1BQY-A	55	23	11	56	44	22	12	11
MER024901	43	24	12	45	55	31	19	12
MER017398	45	24	12	57	43	25	13	12
MER028331	53	23	11	55	45	26	13	13
MER016541	51	20	14	51	49	28	19	9
Mean ± SD	58 ± 9	21 ± 3	11 ± 2	55 ± 5	45 ± 5	21 ± 6	11 ± 4	10 ± 3
**Catalytic core residues**								
MER024901	77	23	9	45	55	23	14	9
MER017398	84	5	5	42	58	16	5	11
MER028331	68	23	18	50	50	18	14	5
MER016541	71	25	8	50	50	21	13	8
Mean ± SD	75 ± 7	19 ± 9	10 ± 6	47 ± 4	53 ± 4	20 ± 3	12 ± 4	8 ± 3

* Amino acid physico-chemical classes: small (Gly, Ala, Ser, Pro, Val, Thr, Cys), aliphatic (Ile, Leu, Val), aromatic (Phe, His, Trp, Tyr), non-polar (Ala, Cys, Phe, Gly, Ile, Leu, Met, Pro, Val, Trp, Tyr), polar (Asp, Glu, His, Lys, Asn, Gln, Arg, Ser, Thr), charged (Asp, Glu, His, Lys, Arg), basic (His, Lys, Arg), and acidic (Asp and Glu).
